# Driving the quadricyclane-to-norbornadiene isomerization by charge separation with perylenediimide as electron acceptor†

**DOI:** 10.1039/d3sc03679k

**Published:** 2023-09-20

**Authors:** Wiebke Zika, Andreas Leng, René Weiß, Simone Pintér, Christoph M. Schüßlbauer, Timothy Clark, Andreas Hirsch, Dirk M. Guldi

**Affiliations:** a Department of Physical Chemistry I, Friedrich-Alexander-Universität Egerlandstraße 3 91058 Erlangen Germany dirk.guldi@fau.de; b Department of Organic Chemistry II, Friedrich-Alexander-Universität Nikolaus-Fiebiger-Straße 10 91058 Erlangen Germany; c Computer Chemistry Center, Friedrich-Alexander-Universität Nägelsbachstraße 25 91052 Erlangen Germany

## Abstract

Through comprehensive photo-assays, this study investigates the reaction coordinate governing the interconversion between quadricyclane (QC) and norbornadiene (NBD) upon photo-irradiation up to a wavelength of 550 nm. To harness this spectroscopic range for energy release, we link the NBD-core with a highly electron-accepting perylenediimide (PDI) with broad absorption, achieving strong electronic coupling between them. We detail the successful synthesis and present extensive DFT calculations to determine the amount of stored energy. By means of transient absorption spectroscopy, an oxidative electron transfer is observed during the QC-to-NBD isomerization following the initial PDI photoexcitation. This charge-separated state is key to triggering the back-isomerization with visible light excitation.

## Abstract 2

The complementary use of time-resolved and steady-state spectroscopies has made it possible to describe the full reaction coordinate regarding the photoisomerization of NBDs featuring electron accepting PDIs.

## Graphical abstract

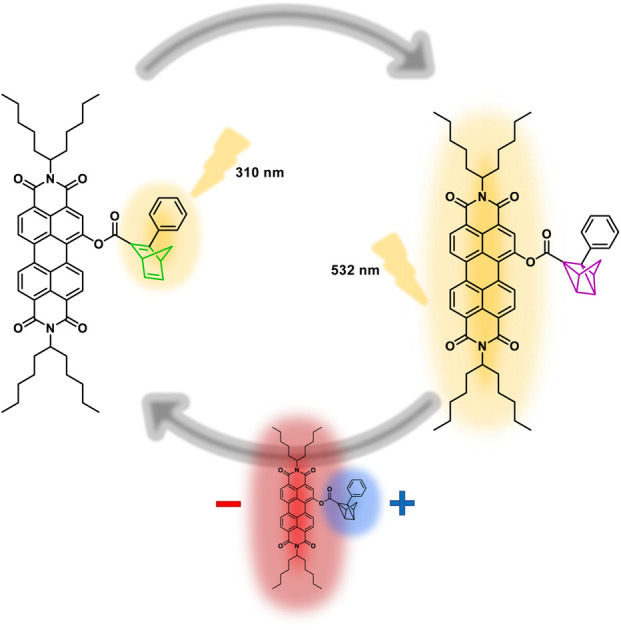

## Introduction

Today's global energy crisis has not only made generating green energy ever more vital, but also placed emphasis on the ways and means of conveniently storing and releasing this energy.^1–4^ The norbornadiene (NBD)/quadricyclane (QC) couple is a blueprint that covers energy conversion, energy storage, and energy release. The earliest reports date back to the 1950s^5,6^ followed by in-depth investigations in the 1960s.^7–10^ The recent past has seen a resurgent interest in the NBD/QC couple given its importance in the field of molecular solar thermal energy storage systems (MOST).^11–19^ It fulfils most of the important MOST criteria. For example, the two photoisomers, NBD and QC, display different ground-state energies and absorb spectrally quite differently from each other. Moreover, the activation barrier for the back-isomerization enables a clean and efficient isomerization under ambient conditions.^20,21^ Its low molecular mass is an asset as it leads to high energy density. In the current context, it is important that the QC oxidation potential is lower than that of NBD.^22–25^ Major drawbacks of the NBD/QC couple stem, however, from their rather poor absorption across the visible region of the solar spectrum. Placing electron-withdrawing and/or electron-donating functionalities at the NBD double bonds by synthetic protocols helps circumvent this drawback. Push–pull effects allow the absorption to be shifted toward longer wavelengths.^11,14,18,26–30^ In a previous study, photoexciting into the long wavelength push–pull absorption at around 400 nm triggered both an efficient NBD-to-QC isomerization and its corresponding QC-to-NBD back-isomerization.^28^ It was important that charge separation did not occur along the reaction profile in the two processes, but rather that a charge transfer mediated both isomerizations. In the current work, we took the aforementioned approach to the next level by replacing the weakly electron-accepting ester with a strongly electron-accepting perylene diimide (PDI). This opened the door to a photochemically driven charge separation to form the one-electron reduced form of PDI next to the one-electron oxidized form of NBD/QC, rather than charge transfer. Unlike the one-electron oxidized form of NBD, which is isomerization-inactive, the one-electron oxidized form of QC catalyzes the regeneration of NBD.^31–35^ PDI photoexcitation in a range up to 550 nm back-isomerizes QC-to-NBD and releases 101 kJ mol^−1^ (Table S2†) in a spectroscopic range much different from where the NBD-to-QC isomerization takes place. Enabling the energy release at 550 nm is a significant step closer to the realization of the NBD/QC couple for practical application, especially when comparing 380 nm in, for example, the naphthalene bisimide (NDI)^19^ case or to 400 nm in our recent work.

## Results and discussion

### Synthesis of NBD–PDI conjugates

Two novel conjugates that differ with respect to their linkers were synthesized, characterized, and investigated in photo-isomerization schemes. One of the two carries a short and rigid linker to connect NBD with PDI, while the second features a long and flexible linker.

**Scheme 1:**
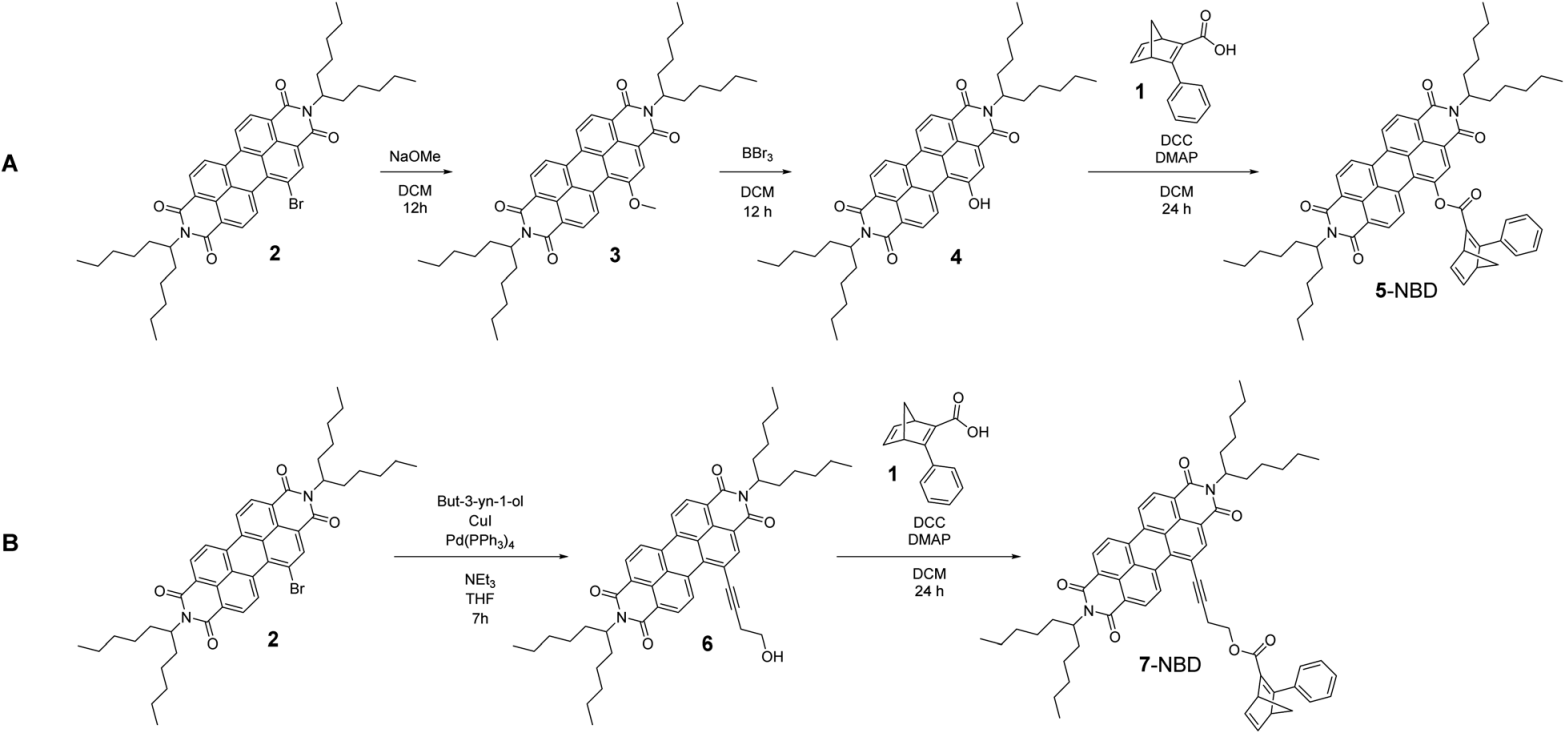
Synthesis of 5-NBD (pathway A) and 7-NBD (pathway B).

For the synthesis of the two target conjugates, NBD 1 was synthesized as a precursor according to a slightly modified literature procedure^36^ based on an ester sonification. Synthesis of the two NBD–PDI conjugates followed the reaction routes sketched in Scheme 1. Synthetic pathway A, leading to the rigidly linked 5-NBD, started with the synthesis of PDI-OMe 3, using PDI-Br 2 as PDI precursor.

In the next step, treatment of the PDI-ether 3 with BBr_3_ led to the corresponding alcohol PDI-OH 4. The final step, which was a Steglich esterification between PDI-OH 4 and the NBD precursor 1, using DCC and DMAP, yielded 5-NBD. The flexibly linked 7-NBD, with its larger distance between PDI and NBD, was synthesized according to reaction of synthetic pathway B. In the first step, a Sonogashira coupling of PDI-Br 2 and but-3-yn-1-ol was performed to obtain the acetylene-bridged alcohol PDI-OH 6. 7-NBD was finally obtained after a Steglich esterification between PDI-OH 6 and NBD precursor 1. Successful syntheses were in both cases confirmed by means of NMR, HRMS, and absorption spectroscopy. All NMR and mass spectra are gathered in the ESI (Fig. S1–S18).†

### Density-functional theory calculations

Density-functional theory (DFT) calculations were used to characterize structures and determine the isomerization enthalpy. First, the isomerization enthalpy was benchmarked in search of a method to model the molecular properties. Here, B3LYP/def2TZVP yielded the best results (Table S1 and Fig. S20†). The PDIs are slightly twisted in the optimized structures (Fig. S21†) because of repulsive interactions across the unsymmetrical bay substitution. In 7-NBD and 7-QC, the presence of an acetylene group induces a slight tilting of NBD/QC relative to PDI. This allows the phenyl group to overlap the PDI centrally, while it is located closer to the PDI edge in 5-NBD and 5-QC. Other than that, no major structural changes are seen between 5-NBD/QC and 7-NBD/QC. The calculated isomerization energy including vibrational zero-point energy corrections is approximately 116 kJ mol^−1^ for 5-NBD/QC and 112 kJ mol^−1^ for 7-NBD/QC (Table S2†). The deviation from literature values of the pristine NBD/QC system (98 kJ mol^−1^)^37^ can be ascribed to substituent effects, which were estimated to 15 and 11 kJ mol^−1^ for 5-NBD and 7-NBD, respectively, using an isodesmic reaction scheme (Scheme S1†). Hence, the actual storage energy for both systems is 101 kJ mol^−1^.

### Steady-state characterization of ground and excited states

**Fig. 1:**
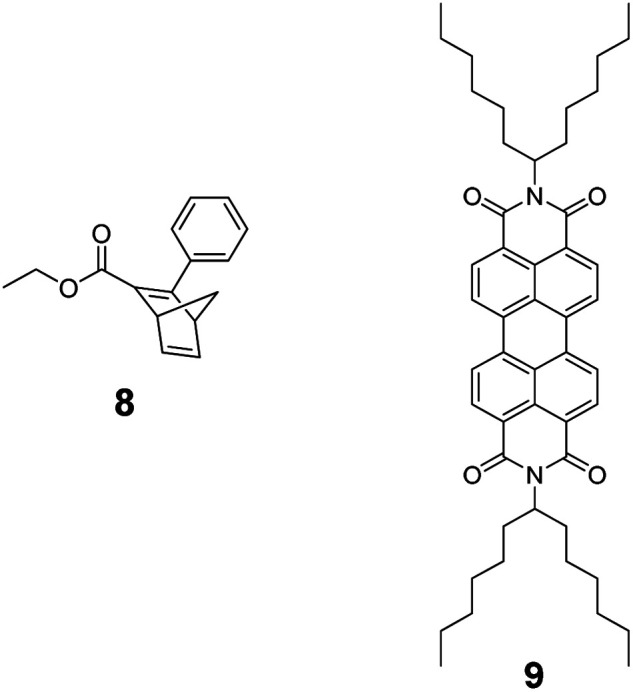
Reference molecules 8 (left) and 9 (right).

**Fig. 2:**
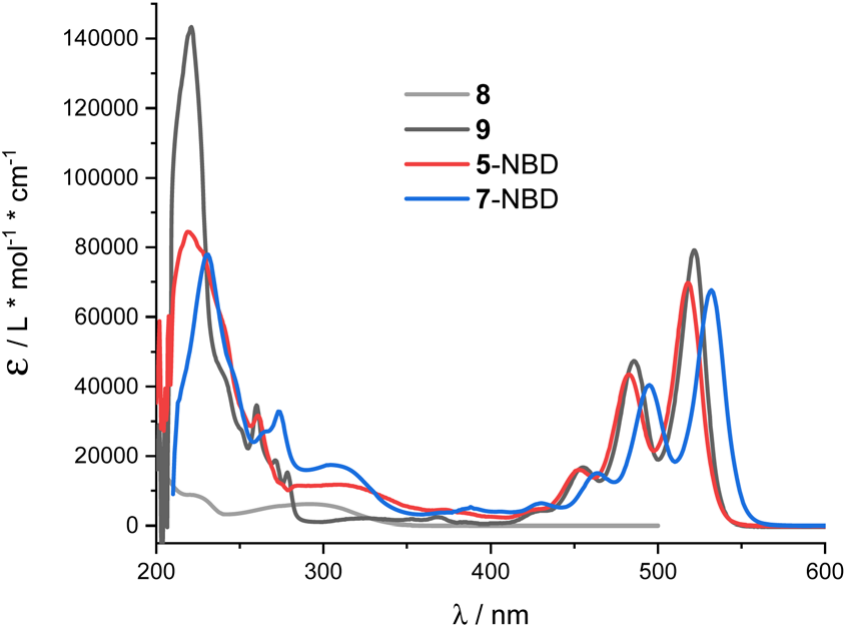
Absorption spectra of 8 (grey), 9 (black), 5-NBD (red) and 7-NBD (blue) in THF.

THF proved to be the solvent of choice based on the NMR photo-isomerization experiments (see below) as it gave the highest interconversion yields. NBD 8 and PDI 9 served as references (Fig. 1). 5-NBD (Fig. 2) exhibits a major maximum at 218 nm in the UV range and a set of four maxima at 425, 453, 483, and 518 nm in the visible range. The absorption between the UV and visible is rather broad and unspecific. The spectroscopic relevant 0–*0 transition at 518 nm is followed by the corresponding 0–*1 transition at 483 nm.^38^ All absorptions across the visible range are slightly blue-shifted compared to PDI 9. Unsymmetrical substitution at the bay position enforces slight out-of-plane twisting and, in turn, a blue-shift.^39,40^

The broad absorption between 280 and 360 nm in 5-NBD is ascribed to NBD, a 30 nm red-shift in comparison with NBD 8. Notably, the strongly electron accepting PDI imparts push–pull character to the NBD double bond. Lastly, the strong absorption at 218 nm, which is largely PDI-centered, is superimposed on the NBD features. For 7-NBD, we find the same absorption features as in 5-NBD, but all PDI-related characteristics are shifted 12 nm bathochromically. Extension of the π-system by the extra acetylene substituent at the bay position is responsible for this shift. In contrast, all NBD-related characteristics, in general, and in particular the 320 nm absorption, remain largely unaffected.

**Fig. 3:**
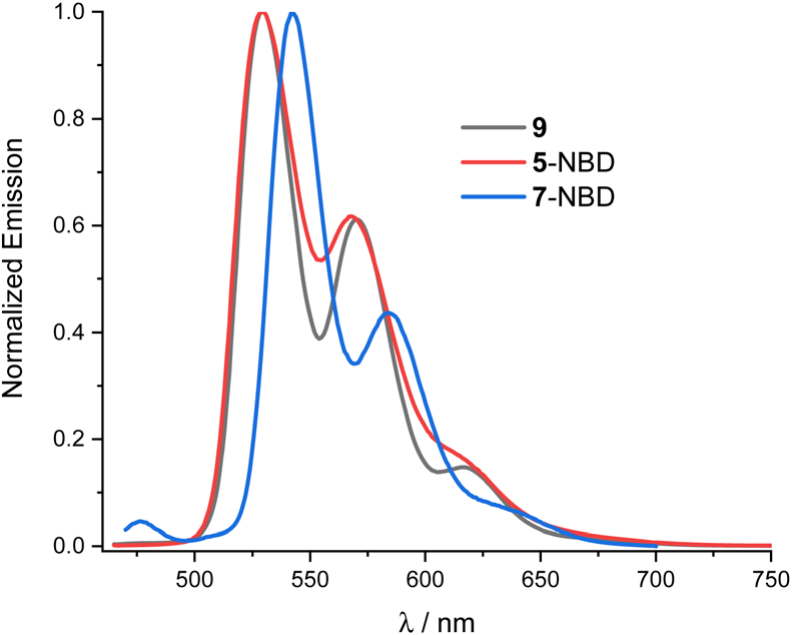
Normalized fluorescence spectra of 9 (black), 5-NBD (red) and 7-NBD (blue) at 430 nm photoexcitation in THF.

For both 5-NBD and 7-NBD, photo-excitation into either the NBD-centered or the PDI-centered absorptions generates the same fluorescence spectrum. This suggests strong electronic communication between PDI and NBD (Fig. S22†). For example, 5-NBD fluoresces with 529 and 568 nm maxima along with 610 and 630 nm shoulders (Fig. 3), giving underlying Stokes-shifts of 11 nm. Compared to PDI 9, a subtle blue shift of 2 nm and an overall broadening are found. Both changes can be rationalized in terms of bay substitution. As for the absorption spectra, the fine-structured fluorescence spectrum of 7-NBD exhibits the same structure as 5-NBD, but is 12 nm red-shifted. For 5-NBD, 7-NBD, and 9, the excitation spectra, taken at the corresponding fluorescence maxima, resemble the corresponding absorption spectra (Fig. S23†).

The PDI fluorescence quantum yields in 9, 5-NBD, and 7-NBD were determined under 310 nm photoexcitation. For 9, the value was 77% in THF. For 5-NBD and 7-NBD, the fluorescence is somewhat quenched with 45% and 35%, respectively. Some, but not all, of the differences are explained by considering the partitioning of the ground-state absorption. The scenario for 5-QC and 7-QC, photoexcited 532 nm rather than 310 nm, is quite different. Neither in 5-QC nor in 7-QC do the QCs exhibit any notable absorption at 532 nm. The corresponding values were 21% and 40% for 5-QC and 7-QC, respectively, and suggest an additional deactivation pathway. The differences in fluorescence quantum yields suggest a higher efficiency for this pathway in 5-QC than in 7-QC. Interestingly, the distance between NBD/QC, on one hand, and PDI, on the other hand, is shorter in the former isomer than in the latter.

In time-correlated single photon counting experiments, the decay curves for 5-NBD, 5-QC, 7-NBD, 7-QC and 9 were all mono-exponential with values in the range from 4 to 5 ns.§§Neither any isomerization nor any switching showed any discernible impact (Table S3 and Fig. S24).

We concluded from differential pulse voltammetry measurements with 5-NBD, 7-NBD, and 9 (Fig. S25†), that all three give PDI-centered reductions at around −1.0 V *vs.* Fc/Fc^+^ (−0.86 V for PDI 9, −1.0 V for 5-NBD, and −1.0 V for 7-NBD) in THF. The underlying difference is explained by the bay-substitution of PDI.^38^ NBD and QC oxidations are reported to take place at +1.17 V *vs.* Fc/Fc^+^ and 0.56 V *vs.* Fc/Fc^+^, respectively.^25^ Taking the one-electron reduction of PDI and the one-electron oxidation of QC into consideration, we estimate an energy of 1.56 eV for the charge-separated state. A charge-separated state involving NBD rather than QC has an energy of 2.27 eV.

Complementary spectro-electrochemical measurements were carried out for 5-NBD and 7-NBD, (Fig. S26†). For 5-NBD, the differential absorption spectra, which were recorded at a potential of −0.7 V *vs.* Fc/Fc^+^ and beyond, feature prominent ground-state bleaching at 451, 483, and 516 nm and absorption maxima at 690 and 750 nm. 7-NBD showed similar results, but the maxima were red-shifted to 710 and 800 nm, respectively. An additional feature at 960 nm is likely due to decomposition.

### Photo-isomerization assays *via* NMR spectroscopy

NBD functionalization with a weakly electron-donating phenyl and a weakly electron-withdrawing ester in 5-NBD and 7-NBD shifts the absorptions bathochromically relative to the parent NBD. Therefore, photoisomerization assays of the interconversion of NBD to the corresponding QC do not need to employ a triplet photosensitizer; 310 nm photoexcitation is sufficient. PDIs absorb at much longer wavelengths, up to 550 nm. We followed the energy-releasing back-isomerization of QC and recovery of NBD by PDI photoexcitation. The underlying reaction mechanism will be discussed below.

**Scheme 2:**
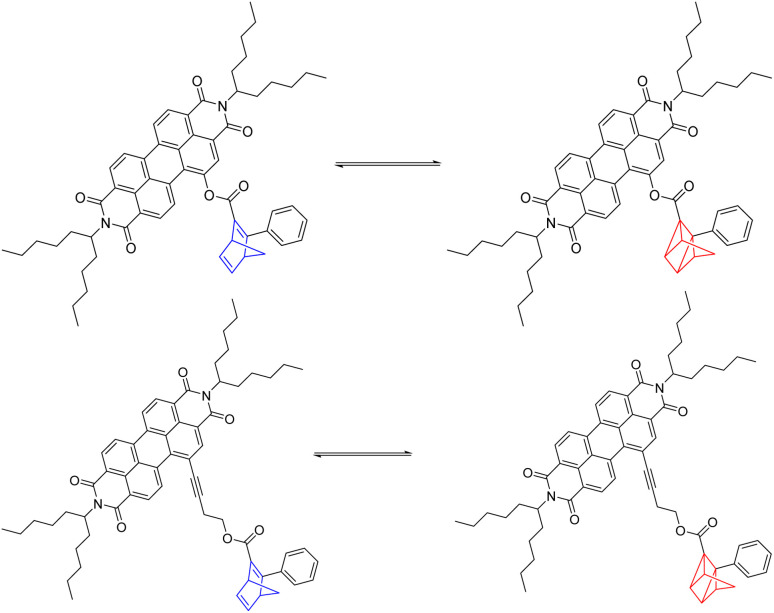
Reversible photoisomerization of 5-NBD to 5-QC on the top and of 7-NBD to 7-QC on the bottom.

To investigate the energy storage ability, photoisomerization experiments (Scheme 2) were performed in solution at a concentration of 7.93 mmol L^−1^. Both NBD–PDI conjugates were isomerized to the corresponding QC by LED irradiation at 310 nm. Back-isomerization by PDI photoexcitation with a 475 nm LED allowed the initial NBDs to be recovered.

The isomerization reactions were followed by NMR spectroscopy and the absolute conversion rates were determined by integrating the NBD- and QC-related signals (for details, see the ESI Fig. S19, S27–S33 and Tables S4–S10†). None of the irradiation assays resulted in a quantitative interconversion between NBD–PDI and QC–PDI, probably because the residual PDI absorption at 310 nm triggers the back-isomerization (see below). A static equilibrium between the two photoisomers is observed after a given irradiation time.

**Fig. 4:**
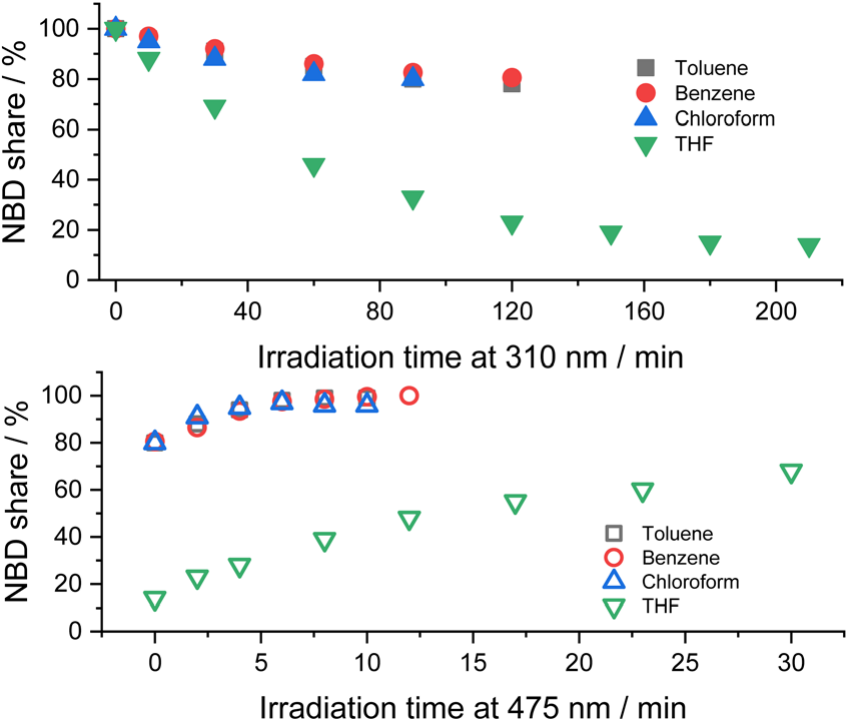
Time progressions of the photoisomerization of 5-NBD to 5-QC (310 nm irradiation, top) and back (475 nm irradiation, bottom) in different solvents.

Initial assays were performed with 5-NBD and 5-QC in solvents of different polarity (Fig. 4). In, for example, chloroform a steady-state equilibrium of 80% 5-NBD and 20% 5-QC was attained after 1.5 h of 310 nm irradiation. 12 min of 475 nm irradiation gave 95% back-conversion with 5% decomposition. In benzene, the overall interconversion, that is, a full cycle of isomerization and back-isomerization under 310 and 475 nm irradiation, was fully reversible. The maximum yield of 5-QC, however, never surpassed 19.5%, even after 2 h of continuous 310 nm irradiation. The 5-QC yield in toluene was slightly higher (25%), but only 99% of 5-NBD was recovered in the back isomerization. By far the highest yields of 5-QC (86% after 3.5 h of irradiation at 310 nm) were obtained using THF as solvent. The decomposition was, however, significant in the subsequent back-isomerization, and only 68% of 5-NBD were recovered, probably because the remaining 32% decomposed under 475 nm irradiation. Despite a 30 seconds purge with argon, which was used for all samples prior to the photoisomerization experiments, leftover traces of O_2_ cannot be ruled out, especially because adding small but known amounts O_2_ increases the decomposition (see below).¶¶At this point, we stopped pursuing any further experiments in chloroform. The combination of poor conversions and significant decompositions rationalized our decision.

**Fig. 5:**
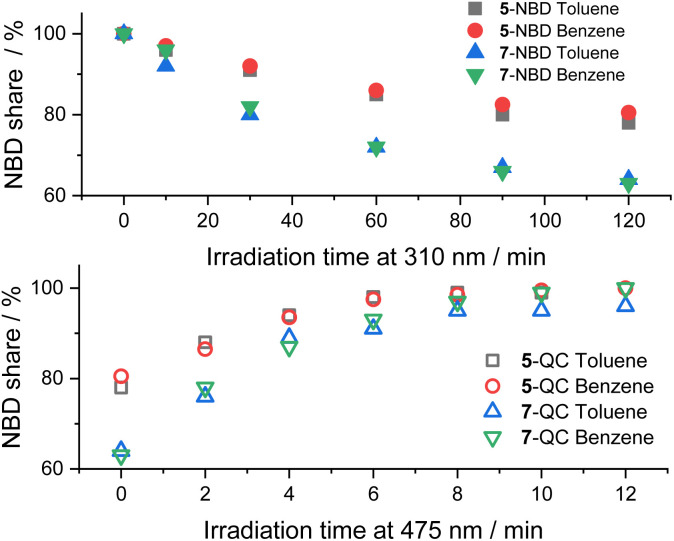
Time progressions of the photoisomerization of 5-NBD to 5-QC and 7-NBD to 7-QC (310 nm irradiation, top) and back (475 nm irradiation, bottom) in toluene and benzene.

In Fig. 5, we compare the photoisomerization experiments for the two NBD–PDI isomers in toluene with those in benzene. 7-NBD gives higher conversion with 36% and 37% yields of 7-QC in toluene and benzene, respectively. Much like the conclusions, drawn for 5-NBD, the overall interconversion for 7-NBD is far from reversible in toluene. Only 96% of 7-NBD were recovered, although the back-isomerization is quantitative in benzene.

**Fig. 6:**
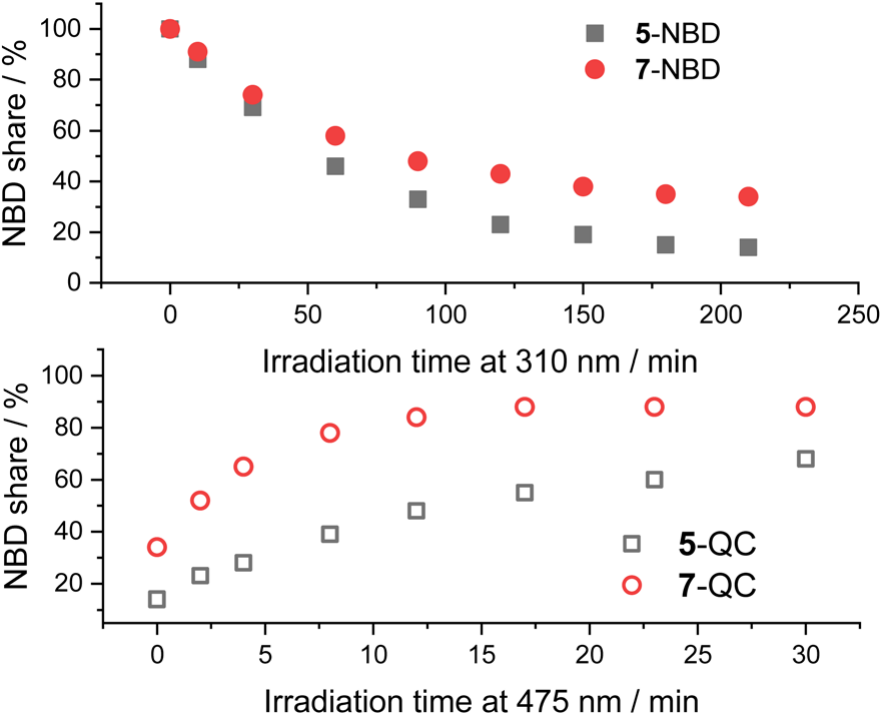
Time progressions of the photoisomerization of 5-NBD to 5-QC and 7-NBD to 7-QC (310 nm irradiation, top) and back (475 nm irradiation, bottom) in THF.

A comparison between the two NBD–PDI isomers in THF is shown in Fig. 6. 310 nm irradiation of 7-NBD gave a 66% NBD-to-QC conversion yield after 3.5 h. The decomposition was, however, considerable throughout the back-isomerization under 475 nm irradiation. The benzene and toluene tests showed little difference between the two (NBD–PDI)s, but the outcome was significantly different when using THF. The yield of 5-QC was much higher at 86%. In summary, all time progressions were similar. The cleanest interconversions were found in benzene. Besides that, no clear trend in terms of conversion was found between the two systems. For example, 5-NBD shows a higher conversion rate in THF than 7-NBD, while the reverse is true in benzene and toluene.

### Photo-isomerization assays by absorption spectroscopy

**Fig. 7:**
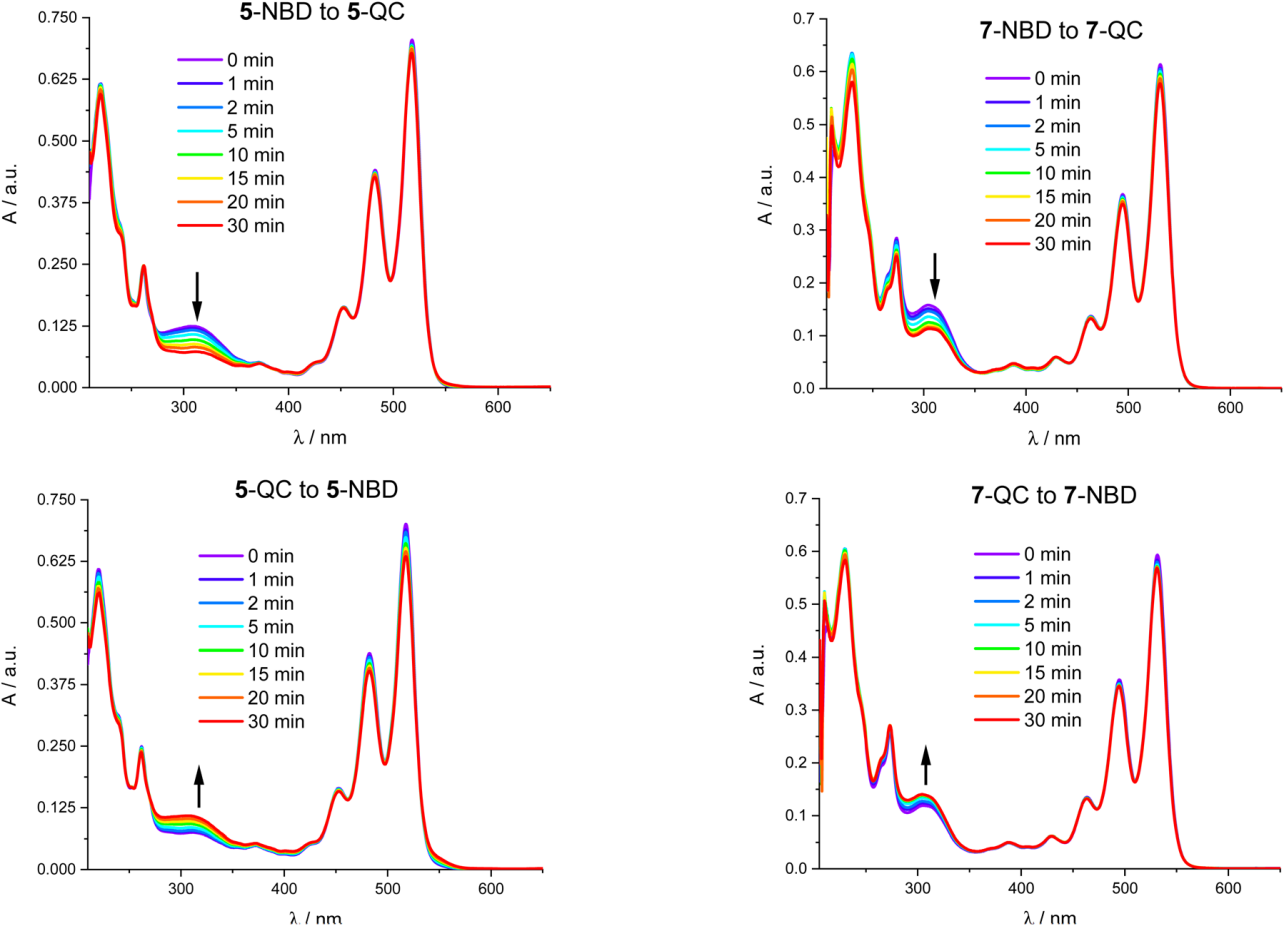
Absorption spectra of 4 × 10^−5^ M 5-NBD in THF between 0 and 30 minutes of photoirradiation at 310 nm (top left), 7-NBD between 0 and 30 minutes of photoirradiation at 310 nm (top right), 5-QC between 0 and 30 minutes of photoirradiation at 532 nm (bottom left), and 7-QC between 0 and 30 minutes of photoirradiation at 532 nm (bottom right).

Fig. 7 shows that 310 nm irradiation of 5-NBD in THF and an Ar atmosphere leads to a 48% decrease in 5-NBD-absorption. Please note that this value does not coincide with the degree of isomerization due to PDI light partitioning at this wavelength. Any differences relative to the NMR experiments can be ascribed to the different concentrations, as has been shown recently.^41^ After subsequent irradiation at 532 nm for 30 minutes (Fig. 7), the absorption re-emerges to 84% of the initial value, which is equivalent to a 32% increase. For 7-NBD (Fig. 7), the decrease in 7-NBD-absorption under identical conditions is only 29% rather than 48% seen for 5-NBD. Of these 29%, only 16% were back-converted to QC with 532 nm irradiation, rendering the QC-to-NBD transformation of 7-QC half as effective as for 5-QC. Analogous experiments carried out in the presence of O_2_ were accompanied by PDI degradation. Most notable is a new absorption feature at 550 nm (Fig. S34†). In 7-QC, oxygen only has a minor influence on the irradiation behavior.

### Transient absorption spectroscopy

Two sets of transient absorption spectroscopic measurements were conducted; on one hand, photoexcitation of 5-NBD at 310 nm and, on the other, photoexcitation of 5-QC and 7-QC at 532 nm. 9 was used as a reference for both sets because 8 lacks any appreciable absorption features beyond 360 nm and is therefore unsuitable for reference measurements.^28^

**Fig. 8:**
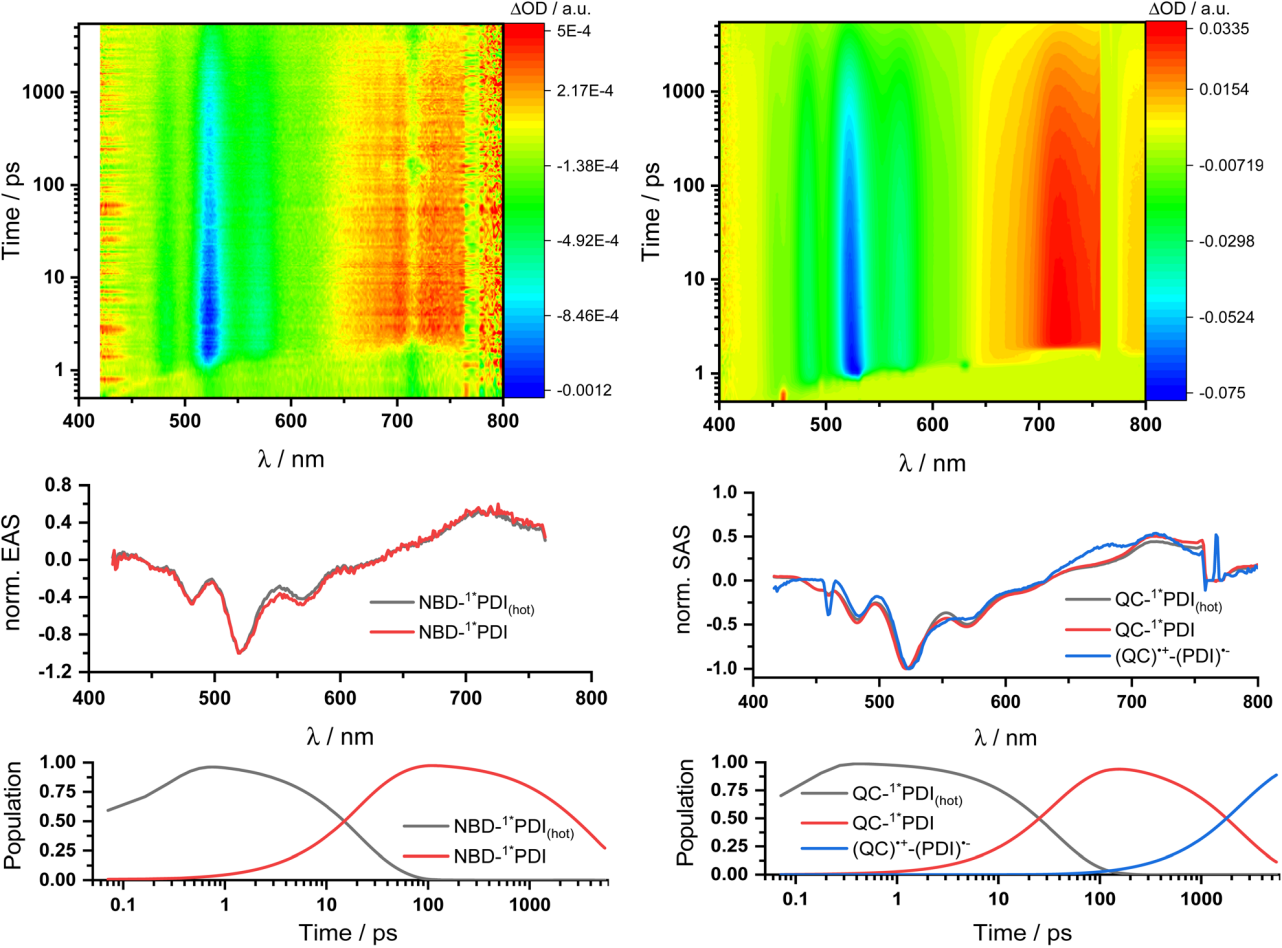
fs-Transient absorption spectroscopy of 1 × 10^−4^ M 5-NBD in THF obtained upon 310 nm photoexcitation on the left and 5-QC upon 532 nm photoexcitation on the right. Top: 3D plots of the raw data. Center: Normalized evolution associated spectra (EAS) on the left and normalized species associated spectra (SAS) on the right obtained by global and target analysis, respectively. Bottom: Population profiles.

**Fig. 9:**
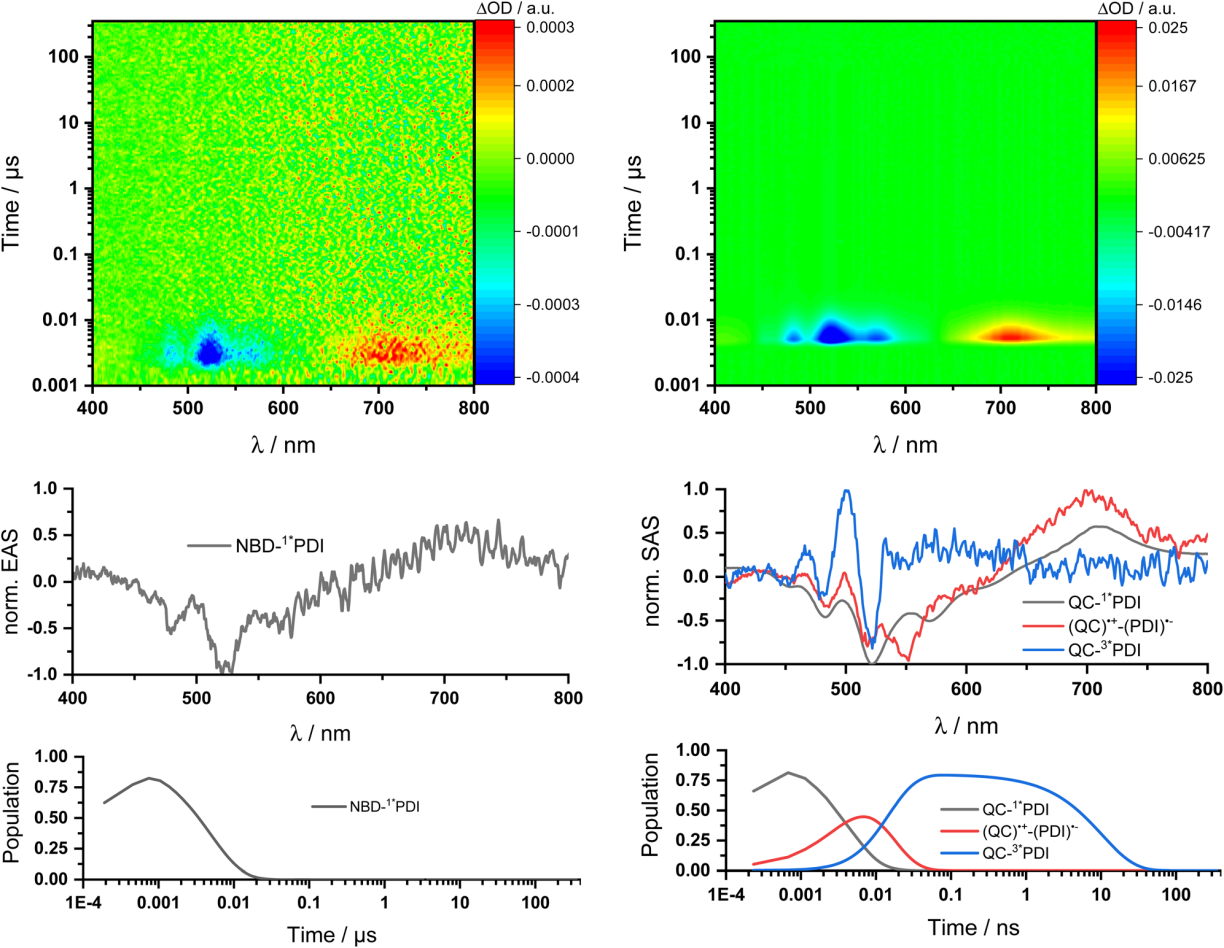
ns-Transient absorption spectroscopy of 1 × 10^−4^ M 5-NBD in THF obtained upon 310 nm photoexcitation on the left and 5-QC upon 532 nm photoexcitation on the right. Top: 3D plots of the raw data. Center: Normalized evolution associated spectra (EAS) on the left and normalized species associated spectra (SAS) on the right obtained by global and target analysis, respectively. Bottom: Population profiles.

Photoexcitation of 5-NBD at 310 nm (Fig. 8 and S37†) leads to the formation of two states, which were best fit with a sequential model. The first species has a lifetime of 21.4 ps and shows prominent ground-state bleaching (GSB) at 482, 520, and 570 nm and excited state absorption (ESA) that maximizes at 720 and 885 nm. The differential absorption features of the second species are quite similar to those of the first, at least with respect to their shape, but they differ significantly in terms of their lifetimes: 4.2 ns. In accordance with the TCSPC measurements (Fig. S24†), we assign the first species to a vibrationally-hot excited state (^1^*PDI_(hot)_) and the second to the fluorescent singlet excited state (^1^*PDI). Turning to ns-TAS (Fig. 9 and S38), we find no further long-lived species next to (1*PDI).

**Fig. 10:**
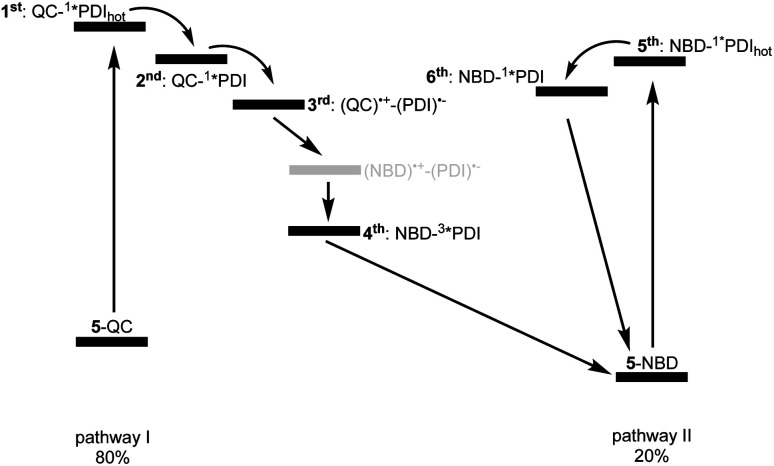
Kinetic model with six different species used to fit the TAS of 5-QC upon 532 nm photoexcitation. Two parallel deactivation pathways, that is, I, on the left, with first, second, third, and fourth species and II, on the right, with fifth, and sixth species, subject to a branching ratio of 80% (5-QC) and 20% (5-NBD). Please note that the (NBD)˙^+^–(PDI)˙^−^ charge separated state in grey is kinetically invisible to us.

Reference experiments conducted with 9 and 310 nm photoexcitation (Fig. S39 and S40†) gave very similar results. Photoexcitation yields (^1^*PDI_(hot)_) as the first species, which decays within 12 ps to afford (^1^*PDI). The lifetime of the latter is 4.34 ns. In the ns-TAS of 9, we failed to find evidence for (^3^*PDI), which is rather typical for unsubstituted PDIs.^42–44^ Overall, we concluded the same reaction sequence from 532 nm photoexcitation experiments with 9 (Fig. S35 and S36†). The only notable difference is a slightly longer lived (^1^*PDI_(hot)_). Its lifetime is 455 ps, to give (^1^*PDI) with a lifetime of 4.46 ns. For the analysis of the fs-TAS experiments with 5-QC following 532 nm photoexcitation (Fig. 8, S41 and S42†), we turned to target analysis. A sequential model could not describe the decay. NMR experiments suggest prolonged photoirradiation of 5-NBD in THF to afford a 5-QC/5-NBD overall ratio of 80% to 20%. We therefore applied a branching model based on two different deactivation pathways, denoted I and II, in order to reflect the parallel photoexcitation of 5-QC and 5-NBD (Fig. 10). This analysis suggests that 80% follow pathway I, in which overall first, second, third, and fourth species were employed in the fitting procedure. The spectroscopic features of the first and second species are identical to those of the fifth and sixth species of pathway II, respectively (see below). All of them involve PDI. Importantly, the short-lived (^1^*PDI_(hot)_) and long-lived (^1^*PDI) of pathway I involve 5-QC with lifetimes of 132 ps and 3.3 ns, respectively. The third species is of great relevance. It evolved from the second species, (^1^*PDI) of 5-QC, and its formation fits the (^1^*PDI) decay perfectly. Importantly, the additional 675 nm ESA agrees well with the fingerprint absorption recorded upon spectro-electrochemical formation of the one-electron reduced form of PDI (Fig. S26†). In other words, a charge-separated state, in which PDI is reduced and QC is oxidized, is formed. Charge separation is thermodynamically driven by 0.84 eV when considering an energy of 2.4 eV for (^1^*PDI) and 1.56 eV for the charge separated state (see above). The charge-separated state lifetime, which was determined in ns-TAS, is 11.6 ns (Fig. 9 and S42†). Beyond this time window, a sixth and final species of the reaction sequence evolves with a 498 nm ESA. Its characteristics fit that of a PDI triplet excited state (^3^*PDI), which is O_2_ sensitive, and has a lifetime is 10 μs. All these data are in sound agreement with the literature.^42,43^ Past experiments have demonstrated that the one-electron oxidized form of QC readily interconverts to the one-electron oxidized form of NBD. Taking this into consideration, we summarize that charge separation is followed by a slow interconversion and a fast charge recombination to populate (^3^*PDI) before the ground state of 5-NBD is recovered. A slow interconversion and a fast charge recombination render the charge-separated state, in which PDI is reduced and NBD is oxidized, invisible. For the remaining 20%, which is due to 5-NBD, we considered pathway II, which proceeds parallel to pathway I. It follows the same deactivation pathway as summarized for the 310 nm photoexcitation experiments with 5-NBD. In particular, a short-lived (^1^*PDI_(hot)_) with a 132 ps lifetime is followed by (^1^*PDI, lifetime 3.26 ns) as fifth and sixth species, respectively. This concludes pathway II with its focus on 5-NBD.

The photoactivity of 7-QC when photoexcited at 532 nm (Fig. S43 and S44†) lies in stark contrast: It is only followed by the PDI-centered (^1^*PDI_(hot)_) and (^1^*PDI) much like what was seen in reference experiments with PDI 9 (Fig. S35 and S36†). Also, contributions of (^3^*PDI) were not seen. The lack of a charge-separated state was puzzling, as photo-isomerization assays with either NMR or UV/vis spectroscopies showed that photoexcitation of 7-QC at 532 nm yields 7-NBD. We also demonstrated that this reaction is less efficient for 7-QC in THF than for 5-QC. Another notable fact is that the photoisomerization of 7-QC to 7-NBD is much less sensitive towards oxygen than for 5-QC (Fig. S34†) and does not involve a loss in FQY. Therefore, we postulate that for 7-QC the interconversion does not proceed *via* a charge-separated state, but *via* an alternative state, which could, for example, involve energy rather than electron transfer. Alternatively, the charge separation contribution is too low in intensity to be detected in transient absorption spectroscopy.

## Conclusion

Two different NBD–PDI conjugates with varying intramolecular distances and their references have been successfully synthesized. In both, NBD photoisomerization to afford the respective QCs and back photoisomerization has been demonstrated to be most effective in THF. In this solvent, the isomerization for 5-NBD and 5-QC was more efficient than for 7-NBD and 7-QC albeit with marked oxygen sensitivity. With the help of TAS and global/target analyses thereof, all states along the reaction sequences of the interconversion were analyzed. Similar pathways were found for 9, 5-NBD, 7-NBD and 7-QC, featuring a vibrationally-hot excited state (^1^*PDI_(hot)_) and a relaxed fluorescent singlet excited state (^1^*PDI). For 5-QC, under PDI rather than NBD or QC irradiation, an additional charge-separated state was observed: its presence not only plays a significant role in the O_2_ sensitivity, but it is also key in the back photoisomerization. 7-QC lacked any charge-separated state. Thus, we have gained important insight into the reversible NBD-to-QC interconversion. Implementation of electron-accepting functionalities opens the door to a photochemically and photophysically driven back-isomerization of the QC photo-isomer to the thermodynamically stable NBD that can be triggered by visible light excitation.

## Data availability

Experimental data are provided in the ESI† or are available from the corresponding author upon request.

## Author contributions

All author contributions are listed in the ESI.†

## Conflicts of interest

The authors declare no conflicts of interest.

## Supplementary Material

SC-014-D3SC03679K-s001
