# Supplementary material for: Driving the quadricyclane-to-norbornadiene isomerization by charge separation with perylenediimide as electron acceptor
Source: Chem Sci. 2023 Sep 20;14(40):11096–104. doi: 10.1039/d3sc03679k (PMC10583742; doi:10.1039/d3sc03679k)
Supplement: SC-014-D3SC03679K-s001 [file SC-014-D3SC03679K-s001.pdf]

Electronic Supplementary Material (ESI) for Chemical Science.
This journal is © The Royal Society of Chemistry 2023

Supporting Information

Driving the Quadricyclane-to-Norbornadiene Isomerization by Charge Separation with Perylenediimide as Electron Acceptor

Wiebke Zika, Andreas Leng, René Weiß, Simone Pintér, Christoph M. Schüßlbauer, Timothy Clark, Andreas Hirsch, and Dirk M. Guldi*

Abstract: Through comprehensive photo-assays, this study investigates the reaction coordinate governing the interconversion between Quadricyclane (QC) and Norbornadiene (NBD) upon photo-irradiation up to a wavelength of 550 nm. To harness this spectroscopic range for energy release, we link the NBD-core with a highly electron-accepting Perylenediimide (PDI) molecule with broad absorption properties, achieving strong electronic coupling between them. We detail the successful synthesis and present extensive DFT calculations to determine the amount of stored energy. By means of transient absorption spectroscopy, an oxidative electron transfer is observed during the QC-to-NBD isomerization following the initial PDI photoexcitation. This charge-separated state is key to triggering the back-isomerization with visible light excitation.

DOI:

Table of Contents

Table of Contents 2
Synthesis 3
Experimental Procedures 16
General Methods 16
NMR Photoswitching Experiments 17
Results and Discussion 18
Computational Details 18
Additional Spectra 21
References 44
Author Contributions 45

Synthesis

Synthesis of NBD 1

According to literature procedure[1] phenylester-NBD (170 mg, 0.701 mmol, 1.0 eq.) and NaOH (576 mg, 14.4 mmol, 9 eq.) were placed in a microwave vial and H_2O (7 mL) and THF (7 mL) were added. After heating for 7 days at 70 °C, H_2O (30 mL) was added to the mixture and the aqueous layer was washed with DCM (3 x 30 mL). Afterwards, the product was precipitated by adding 2N HCl dropwise to the aqueous phase until a pH value of 7 was reached. The formed product was extracted with (DCM (3 x 50 mL) and the solvent was removed in vacuo. NBD precursor **1** was obtained as a white solid (125 mg, 0.590 mmol, 84 %).

^{1}H NMR (400 MHz, $CDCl_3$, 25 °C): δ = 11.92 (*bs*, 1H), 7.53-7.50 (*m*, 2H), 7.39-7.30 (*m*, 3H), 7.01-6.99 (*m*, 1H), 6.92-6.90 (*m*, 1H), 4.09-4.07 (*m*, 1H), 3.87-3.85 (*m*, 1H), 2.28-2.26 (*m*, 1H), 2.09-2.07 (*m*, 1H) ppm (Figure S1).

^{13}C NMR (101 MHz, $CDCl_3$, 25 °C): δ = 170.4, 170.0, 144.0, 140.7, 138.4, 135.5, 129.0, 128.1, 128.0, 70.7, 59.3, 53.0 ppm (Figure S2).

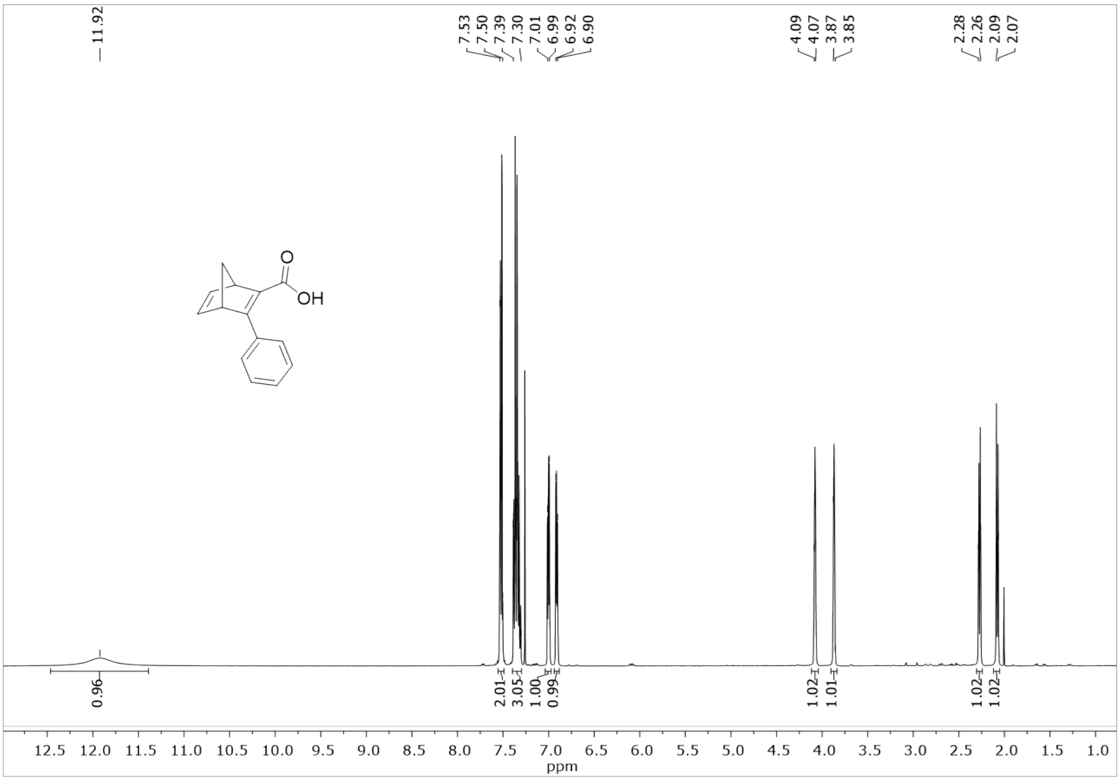

Figure S1. ^{1}H NMR spectrum (400 MHz, rt, $CDCl_3$) of **1**.

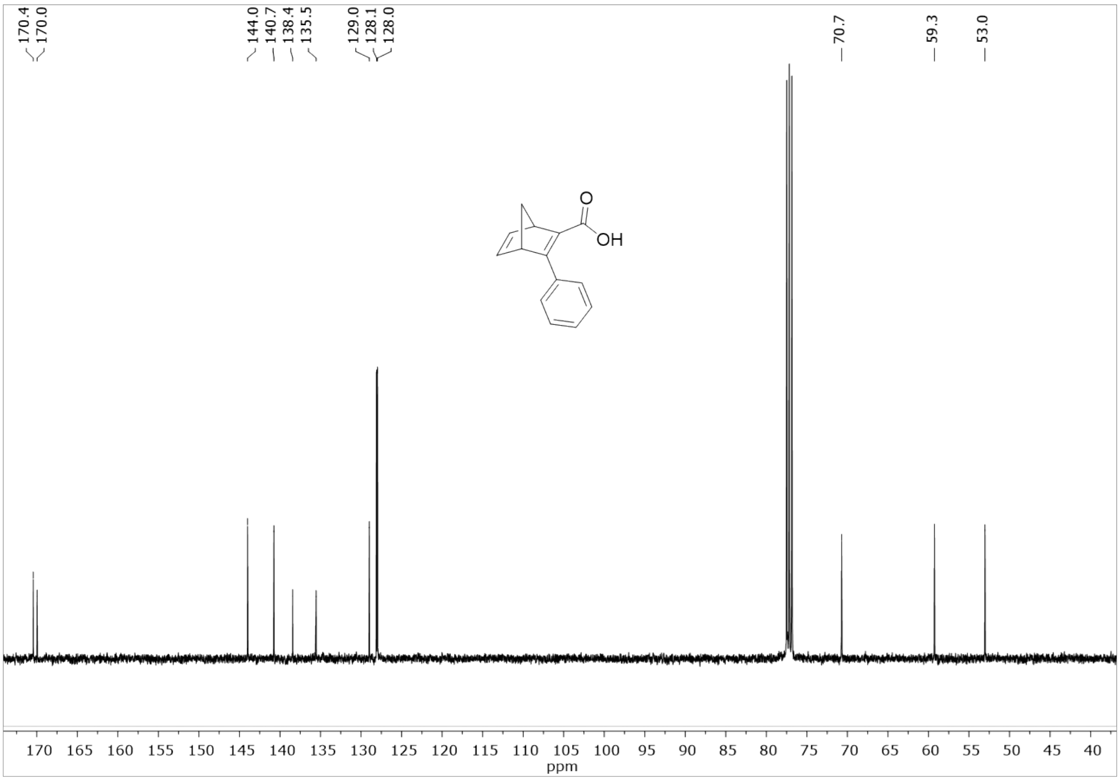

Figure S2. ^{13}C NMR spectrum (101 MHz, rt, $CDCl_3$) of **1**.

Synthesis of PDI-Br 2

According to literature procedure[2] unsubstituted PDI (4.64 g, 6.64 mmol, 1 eq.) was dissolved in DCM (270 mL) and Br_2 (62.4 g, 390 mmol, 20.1 mL, 59 eq.) was added. The reaction was stirred for 41h at room temperature and subsequently quenched, using a saturated aqueous Na_2SO_3 solution (400 mL). The crude product was extracted with DCM (4 x 100 mL) and purified via column chromatography (hexane:DCM v:v 1:2) to obtain title compound **2** in yield of 51% (2.63 g, 3.39 mmol).

^{1}H NMR (400 MHz, $CDCl_3$, 25 °C): δ = 9.80 (*d*, 1H, J = 8 Hz), 8.93 (*bs*, 1H), 8.70 – 8.62 (*m*, 5H), 5.23 – 5.13 (*m*, 2H), 2.30 – 2.19 (*m*, 4H), 1.90 – 1.80 (*m*, 4H), 2.30 – 2.19 (*m*, 4H), 1.37 – 1.23 (*m*, 24H), 0.89 – 0.82 (*m*, 12H) ppm (Figure S3).

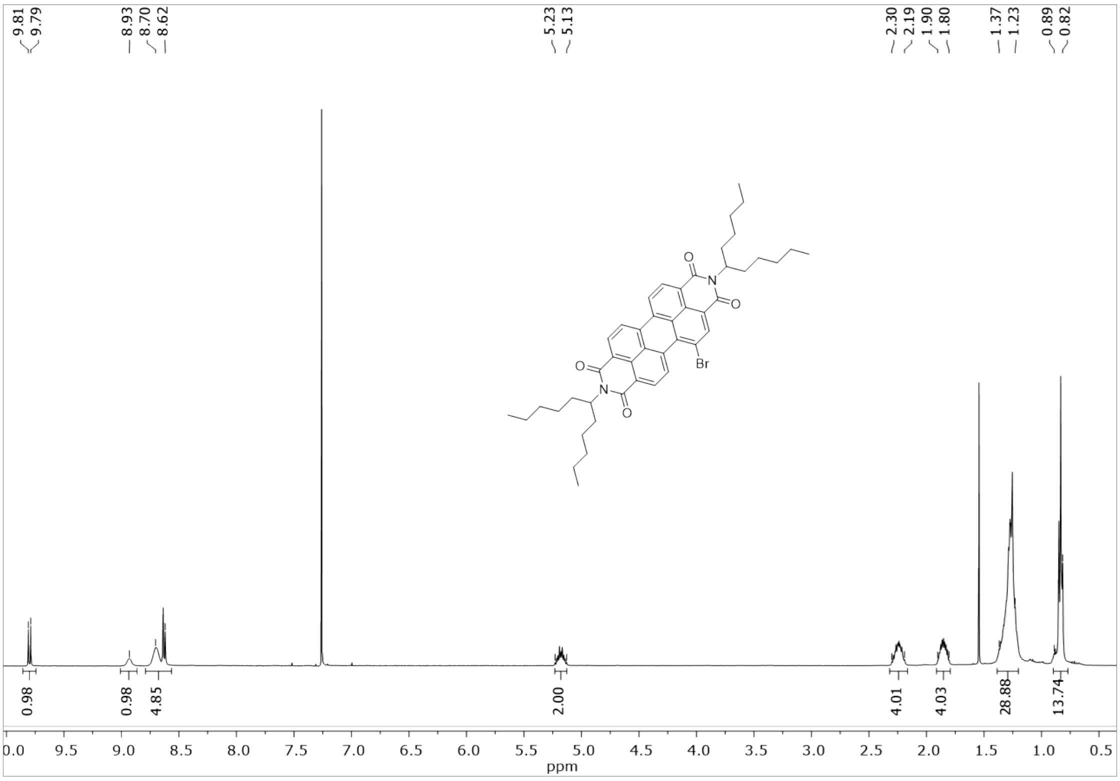

Figure S3. ^{1}H NMR spectrum (400 MHz, rt, $CDCl_3$) of **2**.

Synthesis of PDI-OMe 3

PDI-Br **2** (500 mg, 0.644 mmol, 1 eq.) was dissolved in DCM (20 mL) and a solution of NaOMe (209 mg, 3.86 mmol, 6 eq.) in MeOH (2 mL) was added. After stirring of the reaction overnight, the phases were separated, and the organic layer was washed with 2N HCl (2 x 150 mL) and BRINE (2 x 100 mL) and dried over $MgSO_4$. Crude product was purified via flash chromatography (hexane:DCM v:v 3:7) to obtain title compound PDI-OMe **3** as a dark red solid in a yield of 59% (277 mg, 0.380 mmol).

^{1}H NMR (400 MHz, $CDCl_3$, 25°C) δ = 9.41 (*d*, 1H, J = 8 Hz), 8.60 (*bs*, 2H), 8.49 – 8.42 (*m*, 4H), 5.24 – 5.14 (*m*, 2H), 2.32 – 2.19 (*m*, 2H), 1.93 – 1.84 (*m*, 2H), 1.42 – 1.22 (*m*, 24H), 0.89 – 0.82 (*m*, 12H) ppm (Figure S4).

^{13}C NMR (101 MHz, $CDCl_3$, 25°C) δ = 164.8, 163.8, 158.4, 134.5, 134.4, 134.0, 132.4, 131.6, 131.1, 130.3, 129.3, 129.0, 128.60, 128.5, 127.0, 124.5, 123.9, 123.5, 122.9, 121.9, 120.8, 118.1, 117.5, 57.0, 55.0, 54.8, 32.5, 31.9, 31.9, 26.8, 22.7, 14.2 ppm (Figure S5).

HRMS (APPI, DCM) [M^+H^+] m/z: 729.4262 (calc.), 729.4274 (found).

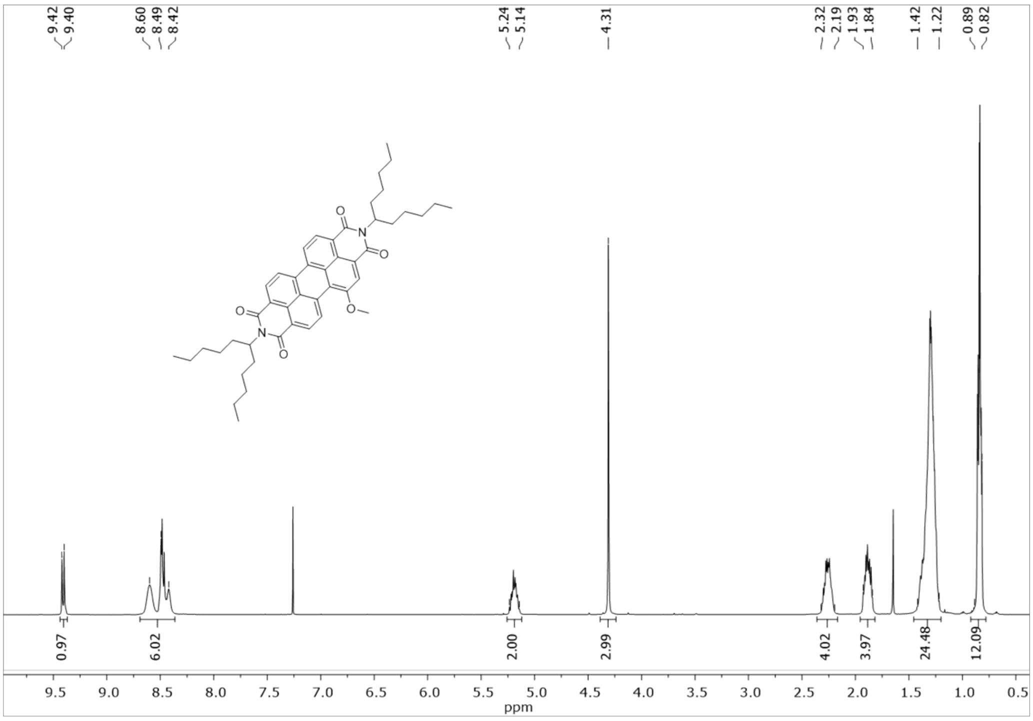

Figure S4. ^{1}H NMR spectrum (400 MHz, rt, $CDCl_3$) of **3**.

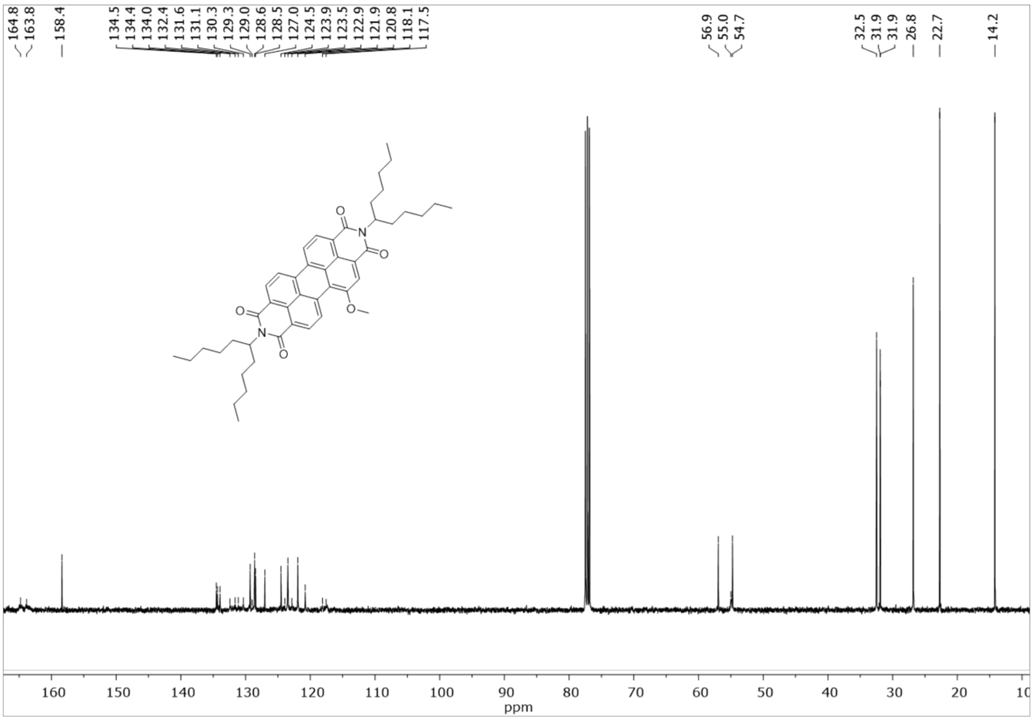

Figure S5. ^{13}C NMR spectrum (101 MHz, rt, $CDCl_3$) of **3**.

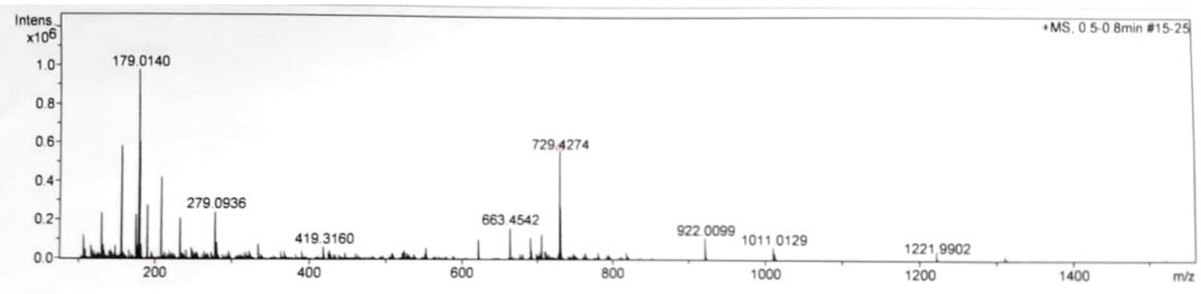

Meas. m/z	#	Ion Formula	m/z	err [ppm]	mSigma	# mSigma	Score	rdb	e⁻ Conf	N-Rule
729.4274	1	C47H57N2O5	729.4262	-1.6	78.1	1	100.00	20.5	even	ok

Figure S6. Mass spectrum of **3** In DCM.

Synthesis of PDI-OH 4

Compound **3** (240 mg, 0.329 mmol, 1 eq.) was dissolved in DCM (10 mL) under N_2-atmosphere and cooled down to 0 °C. Subsequently neat BBr_3 (1.0 M in hexane; 5.92 mmol, 5.92 mL, 18 eq.) was added dropwise and the solution was stirred overnight at RT. Subsequently, the solution was cooled down to 0 °C and methanol was added dropwise, whereby the formed HBr was transferred under a nitrogen flow to a saturated aqueous solution of sodium hydroxide (50 mL). When no reaction of the transferred HBr in the sodium hydroxide solution can be observed anymore, the product solution mixture was poured onto an ice/water mixture. When the mixture reached RT, the organic layer was separated, and the aqueous phase was extracted with DCM (3 x 20 mL). The combined organic layers were washed with a saturated bicarbonate solution (100 mL) and dried over Na_2SO_4. The crude product was purified via column chromatography (DCM:EtOAc, v:v, 99.5:0.5) to obtain the product as a purple solid with a yield of 82% (193 mg, 0.270 mmol).

^{1}H NMR (400 MHz, $C_2D_2Cl_4$, 90°C) δ = 9.51 (*bs*, 1H), 8.62 – 8.43 (*m*, 7H), 5.19 – 5.11 (*m*, 2H), 2.28 – 2.16 (*m*, 4H), 1.93 – 1.87 (*m*, 4H), 1.41 – 1.22 (*m*, 24H), 0.88 – 0.76 (*m*, 12H) ppm (Figure S6).

^{13}C NMR (101 MHz, $CDCl_3$, 25°C) δ = 164.6, 164.0, 156.0, 134.0, 133.6, 132.3, 131.5, 130.9, 130.1, 128.9, 128.4, 128.40, 128.2, 126.3, 124.0, 122.9, 122.7, 122.2, 121.7, 118.8, 55.9, 54.8, 32.4, 31.8, 31.6, 29.7, 26.8, 26.6, 22.7, 22.5, 22.4, 14.1, 13.9 ppm (Figure S7).

HRMS (APPI, DCM) [M^+H^+] m/z: 715.4105 (calc.), 715.4115 (found).

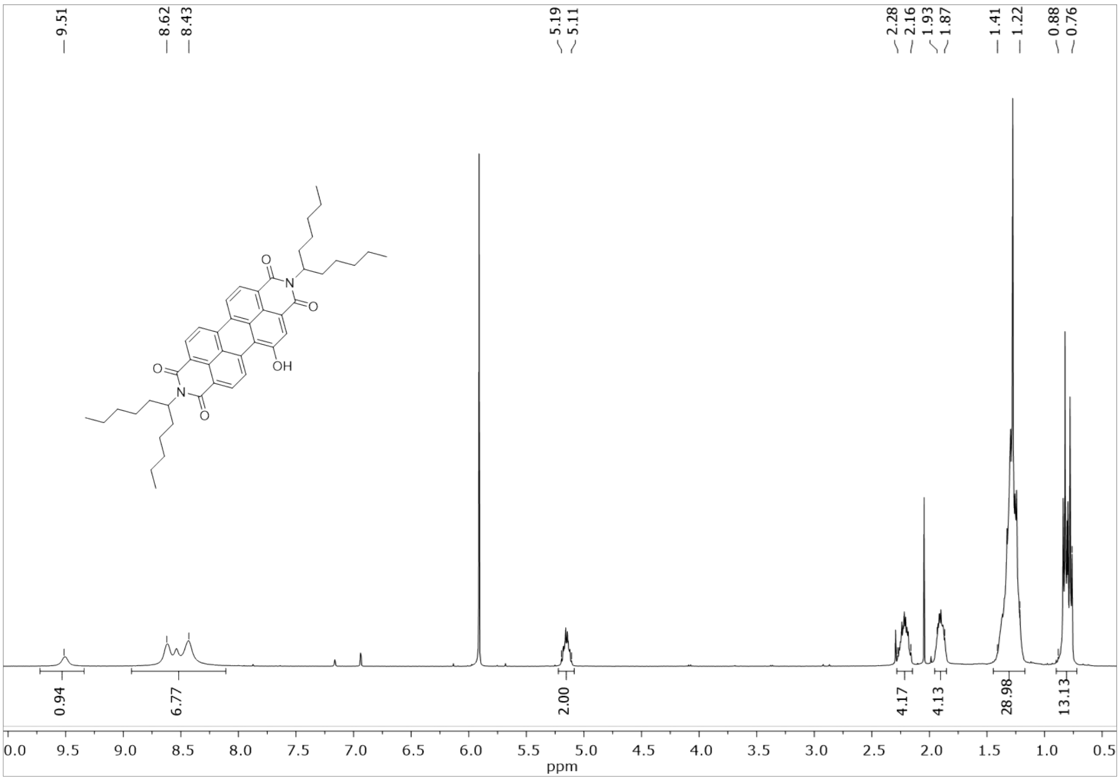

Figure S7. ^{1}H NMR spectrum (400 MHz, 90°C, $C_2D_2Cl_4$) of **4**.

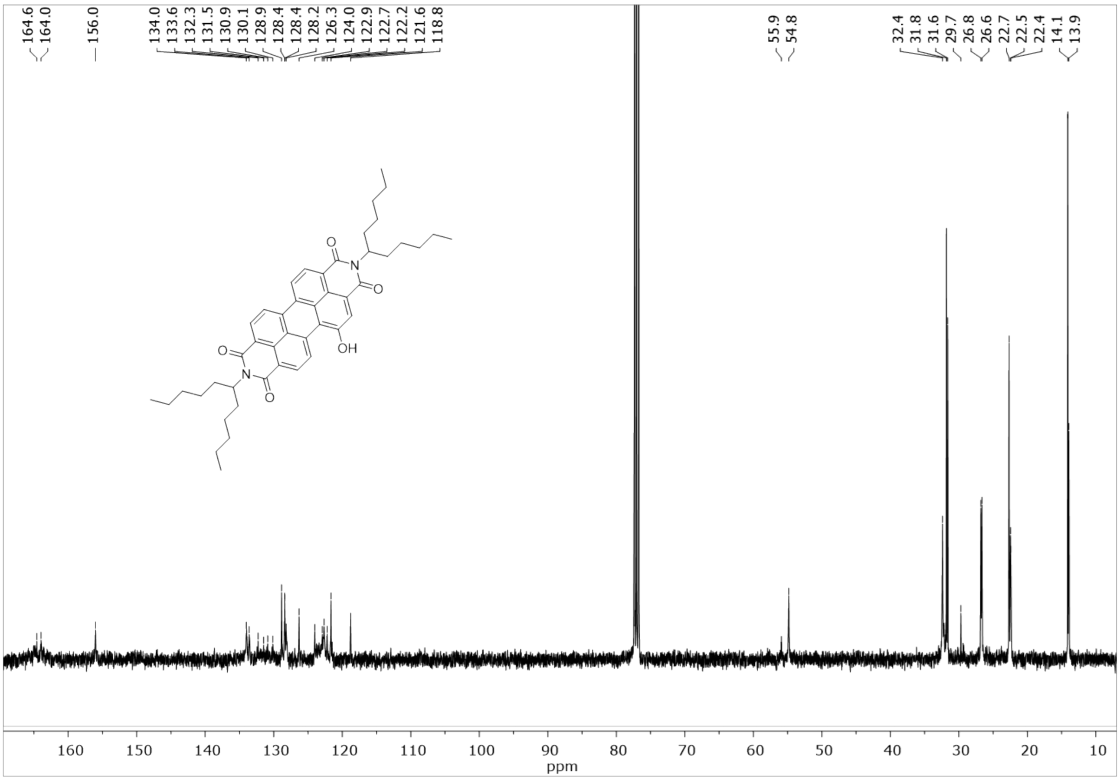

Figure S8. ^{13}C NMR spectrum (101 MHz, rt, $CDCl_3$) of **4**.

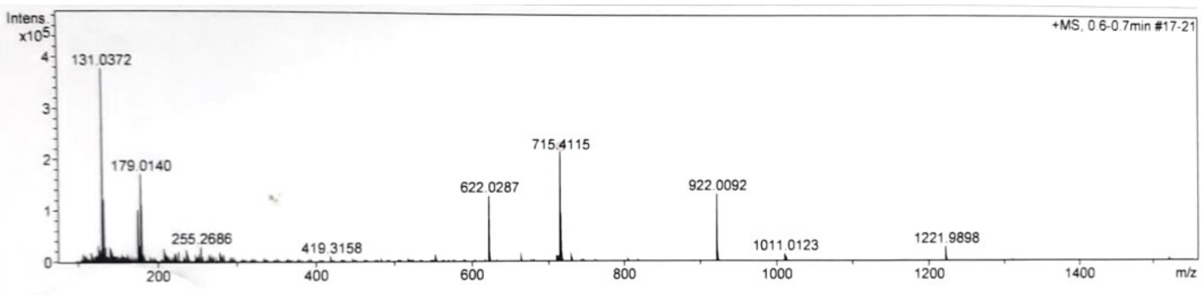

Meas. m/z	#	Ion Formula	m/z	err [ppm]	mSigma	# mSigma	Score	rdb	e⁻ Conf	N-Rule
715.4115	1	C46H55N2O5	715.4105	-1.4	40.4	1	100.00	20.5	even	ok

Figure S9. Mass spectrum of **4** in DCM.

Synthesis of 5-NBD

NBD-Precursor **1** (14.8 mg, 0.0699 mmol, 1 eq.) and DCC (14.4 mg, 0.0699 mmol, 1 eq.) were dissolved in DCM (5 mL) and cooled down to 0°C. Subsequently PDI-OH **4** (50 mg, 0.0699 mmol, 1 eq.) and DMAP (14.4 mg, 0.0699 mmol, 1 eq.) were added and the reaction was stirred for 24h. Subsequently, the reaction mixture was washed with H_2O (50 mL) and BRINE (2 x 50 mL) and dried over $MgSO_4$. The crude product was purified by flash chromatography (Hexane:DCM v:v 1:1) to receive **5**-NBD as a light red solid (50.2 mg, 0.0552 mmol, 79 %).

^{1}H NMR (400 MHz, DCM, 25°C) δ = 8.77 - 8.75 *(m,* 1H), 8.60 – 8.58 (*m*, 4H), 8.39 – 8.33 (*m*, 2H), 7.67 – 7.64 (*m*, 2H), 7.38 – 7.33 (*m*, 2H), 7.31 – 7.27 (*m*, 2H), 7.21 – 7.19 (*m*, 1H), 5.20 – 5.10 (*m*, 2H), 4.47 (*s*, 1H), 4.09 (*s*, 1H), 2.56 – 2.54 (m, 2H), 2.34 – 2.32 (*m*, 2H), 2.29 – 2.18 (*m*, 4H), 1.87 – 1.81 (*m*, 4H), 1.40 – 1.19 (*m*, 24H), 0.87 – 0.82 (*m*, 12H) ppm (Figure S8).

^{13}C NMR (101 MHz, DCM, 25°C) δ = 173.7, 162.5, 148.7, 143.7, 141.4, 137.5, 135.2, 134.4, 134.0, 132.8, 132.8, 131.1, 130.7, 130.0, 129.4, 129.1, 128.6, 128.2, 127.9, 127.9, 127.4, 127.3, 126.7, 125.9, 123.5, 122.8, 71.3, 59.6, 54.7, 54.5, 53.9, 53.6, 53.4, 32.3, 31.8, 31.8, 26.6, 26.6, 22.6, 13.8, 13.8 ppm (Figure S9).

HRMS (ESI, DCM) for $C_{60}H_{64}N_2O_6$ (M^-), calc.: 908.4764, found: 908.4788.

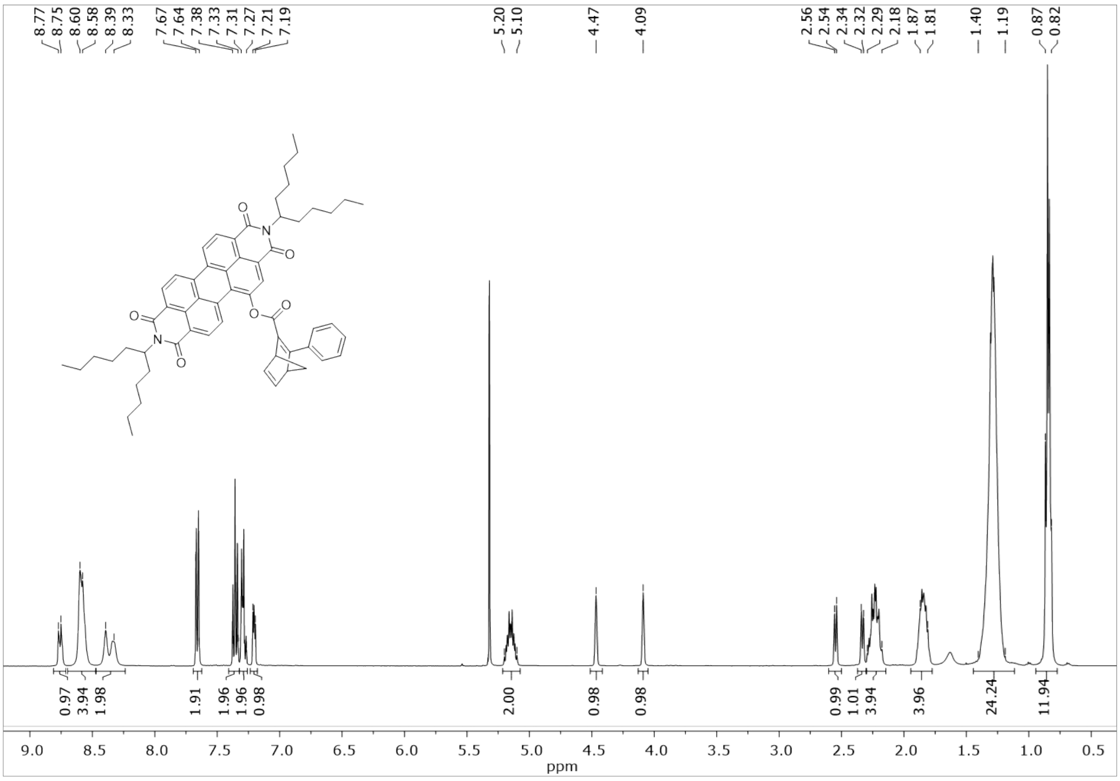

Figure S10. ^{1}H NMR spectrum (400 MHz, rt, CD_2Cl_2) of **5**-NBD.

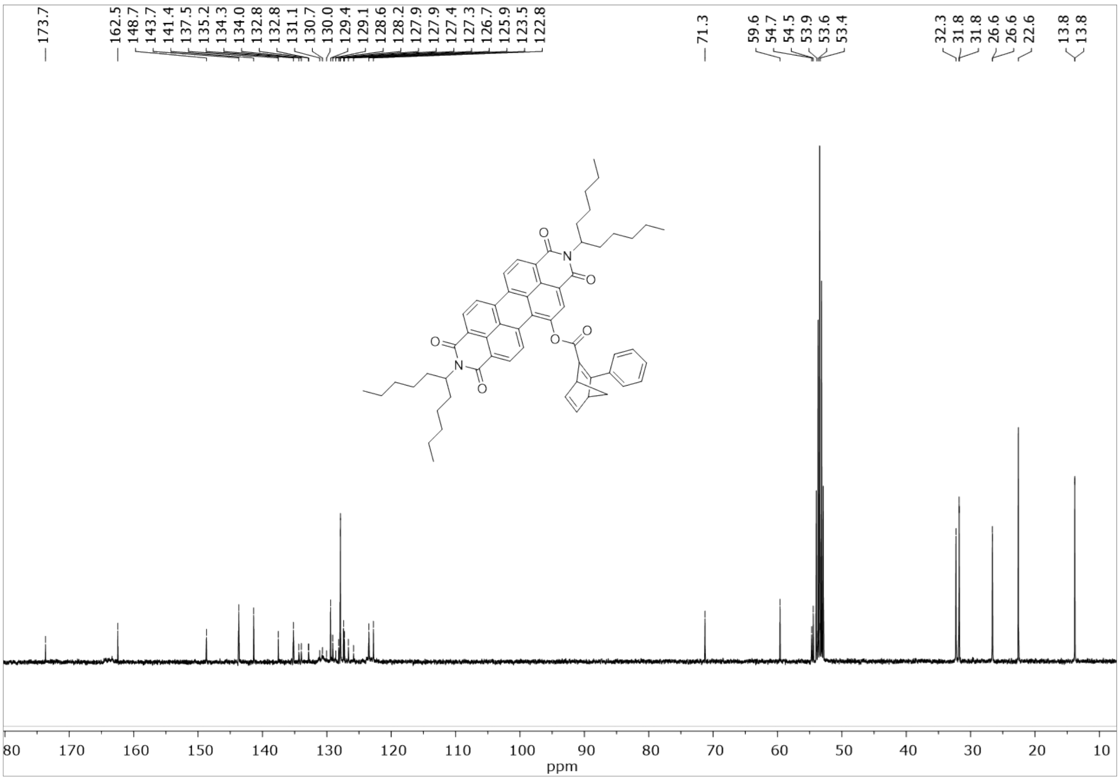

Figure S11. ^{13}C NMR spectrum (101 MHz, rt, CD_2Cl_2) of **5**-NBD.

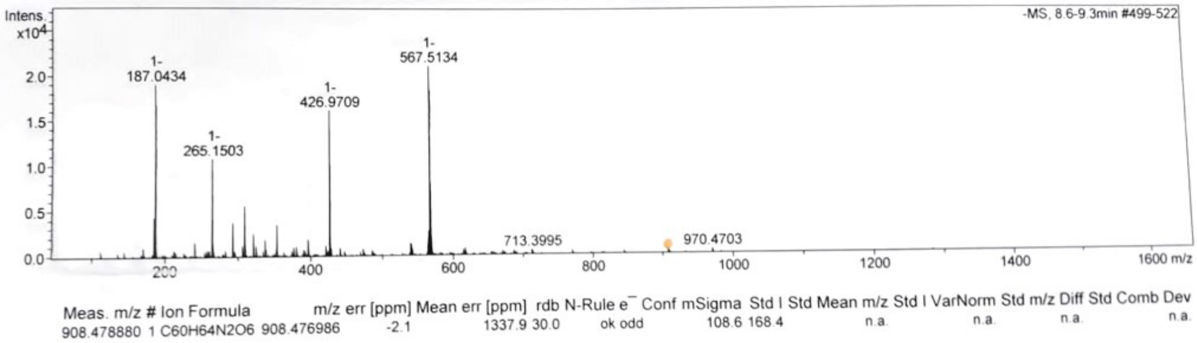

Figure S12. Mass spectrum of **5**-NBD in DCM.

Synthesis of PDI-OH 6

Compound **2** (50 mg, 0.0644 mmol, 1eq.), But-3-yn-1-ol (9.04 mg, 0.129 mmol, 2 eq.), CuI (2.46 mg, 0.0129 mmol, 0.2 eq.) and $Pd(PPh_3)_4$ (7.44 mg, 0.00644 mmol, 0.1 eq.) were dissolved in THF (2 mL) and degassed with argon for 5 min. Subsequently NEt_3 (1 mL) was added, and the reaction was stirred at RT for 7h. Afterwards, the reaction mixture was washed with BRINE (3 x 50 mL) and dried over Na_2SO_4. The crude product was purified via column chromatography ($CHCl_3$:EtOAc v:v 99:1) and PDI-OH **6** was received as a red solid (37 mg, 0.0482 mmol, 75 %)

^{1}H NMR (400 MHz, $CDCl_3$, 25°C) δ = 10.09 (*d*, J = 8 Hz, 2H), 8.56 – 8.44 (*m*, 4H), 8.28 – 8.23 (*m*, 2H), 5.20 – 5.12 (*m*, 2H), 4.10 – 4.06 (*dd*, 2H, J = 8 Hz, 4 Hz), 2.99 - 2.96 (*t*, 2H, J = 6 Hz), 2.77 – 2.74 (*t*, 1H, J = 5.6 Hz), 2.30 - 2.21 (*m*, 4H), 1.93 – 1.87 (*m*, 4H), 1.41 – 1.25 (*m*, 4H), 0.87 – 0.84 (*m*, 12H) ppm (Figure S10).

^{13}C NMR (101 MHz, $CDCl_3$, 25°C) δ = 163.4, 133.9, 133.8, 133.6, 133.3, 133.2, 130.9, 130.3, 128.8, 128.1, 126.7, 126.6, 126.1, 122.9, 122.3, 120.3, 100.1, 83.81, 60.85, 54.9, 54.8, 32.3, 32.3, 31.8, 26.7, 24.7, 22.6, 14.1 ppm (Figure S11).

HRMS (MALDI, DCTB) for $C_{50}H_{58}N_2O_5$ (M^+), calc.: 766.4346, found: 766.4340.

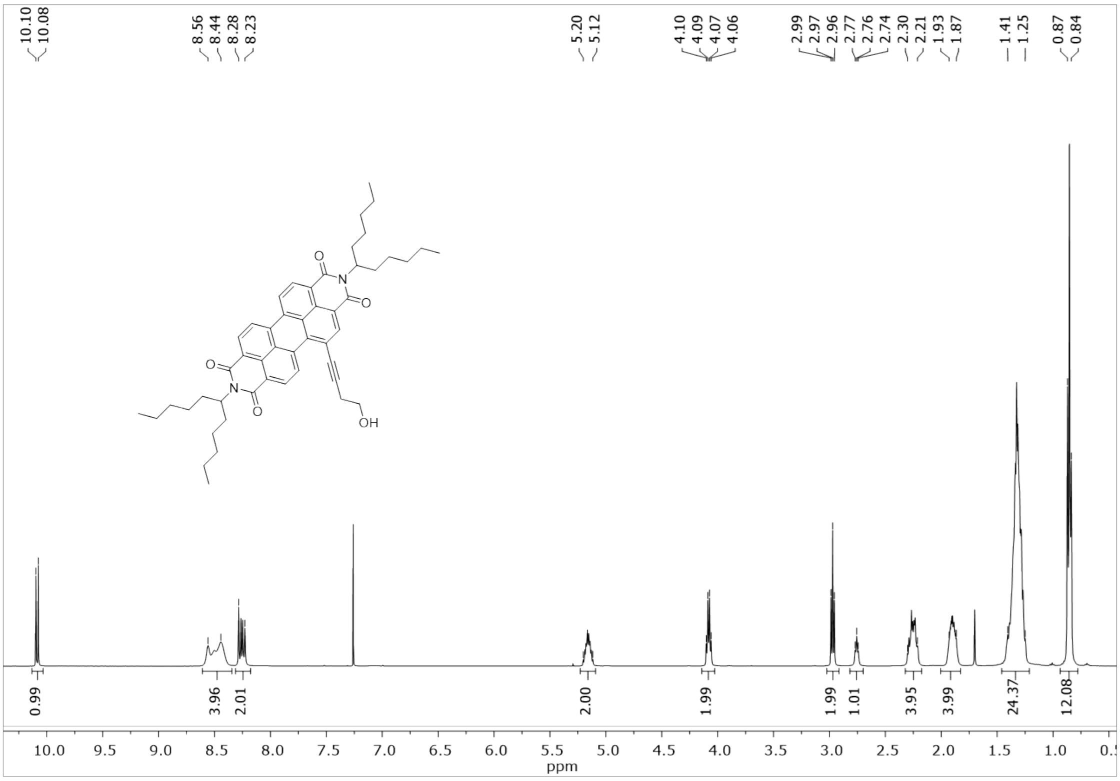

Figure S13. ^{1}H NMR spectrum (400 MHz, rt, $CDCl_3$) of **6**.

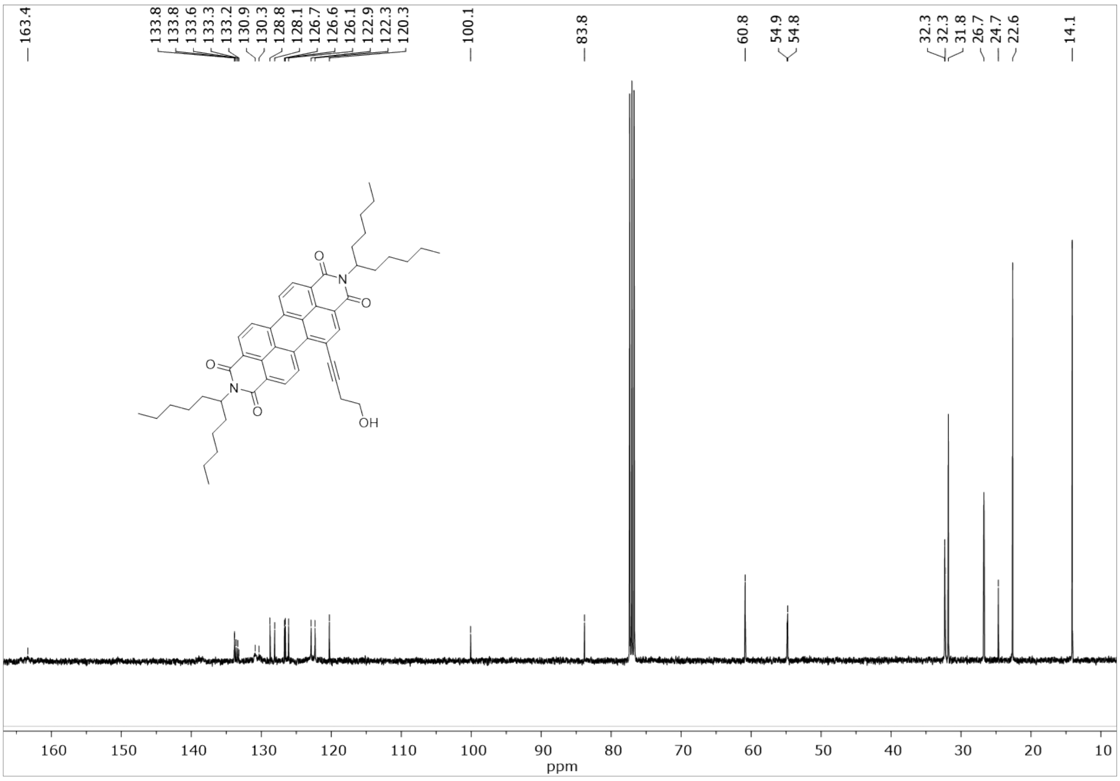

Figure S14. ^{13}C NMR spectrum (101 MHz, rt, $CDCl_3$) of **6**.

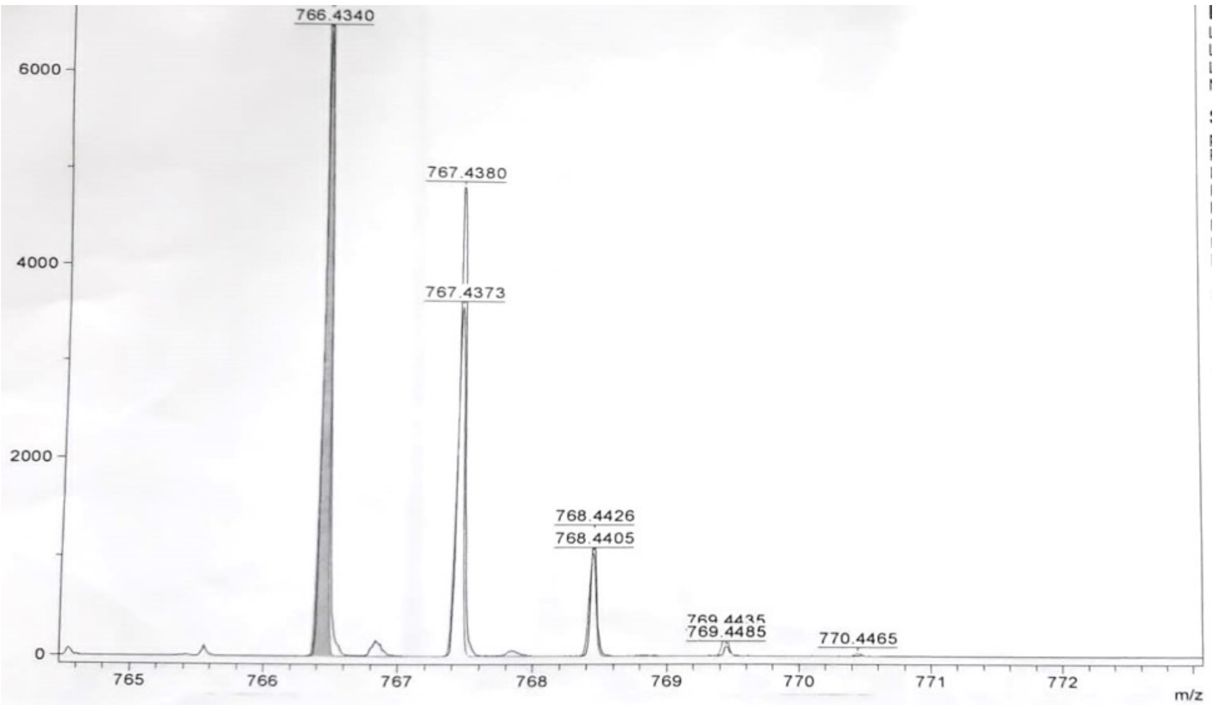

Figure S15. Mass spectrum of **6** in DCM.

Synthesis of 7-NBD

NBD-Precursor **1** (10.2 mg, 0.0482 mmol, 1 eq.) and DCC (9.95 mg, 0.0482 mmol, 1 eq.) were dissolved in DCM (5 mL) and cooled down to 0°C. Subsequently compound **6** (37 mg, 0.0482 mmol, 1 eq.) and DMAP (5.89 mg, 0.0482 mmol, 1 eq.) were added and the reaction was stirred for 24h. Subsequently, the reaction mixture was washed with H_2O (50 mL) and BRINE (2 x 50 mL) and dried over $MgSO_4$. The crude product was purified by flash chromatography ($CHCl_3$ 99:1 EtOAc) to receive **7**-NBD as a red solid (32.4 mg, 0.0337 mmol, 70 %).

^{1}H NMR (400 MHz, $CDCl_3$, 25°C) δ = 10.14 (*d*, J = 8 Hz, 1H), 8.70 – 8.63 (*m*, 6H), 7.51 – 7.48 (*m*, 2H), 7.30 – 7.25 (*m*, 2H), 7.22 – 7.18 (*m*, 1H), 6.99 – 6.97 (*m*, 1H), 6.88 – 6.86 (*m*, 1H), 5.23 – 5.14 (*m*, 2H), 4.45 (*t*, J = 6.7 Hz, 2H), 4.13 (*s*, 1H), 3.82 (*s*, 1H), 2.98 (*t*, J = 6.8 Hz, 2H), 2.31 – 2.21 (*m*, 5H), 2.05 – 2.02 (*m*, 1H), 1.90 – 1.82 (*m*, 4H), 1.38 – 1.22 (*m*, 24H), 0.85 – 0.81 (*m*, 12H) ppm (Figure S12).

^{13}C NMR (101 MHz, $CDCl_3$, 25°C) δ = 168.3, 165.2, 143.9, 140.9, 138.8, 135.8, 134.5, 134.4, 134.0, 129.2, 128.7, 128.5, 127.9, 127.8, 127.3, 126.8, 126.8, 123.5, 123.0, 120.5, 97.7, 83.8, 70.7, 61.6, 58.9, 54.9, 54.8, 53.2, 32.5, 32.4, 31.9, 31.9, 26.8, 26.7, 22.7, 21.0, 14.2 ppm (Figure S13).

HRMS (APPI, DCM) [M^+H^+] m/z: 961.5150 (calc.), 961.5160 (found).

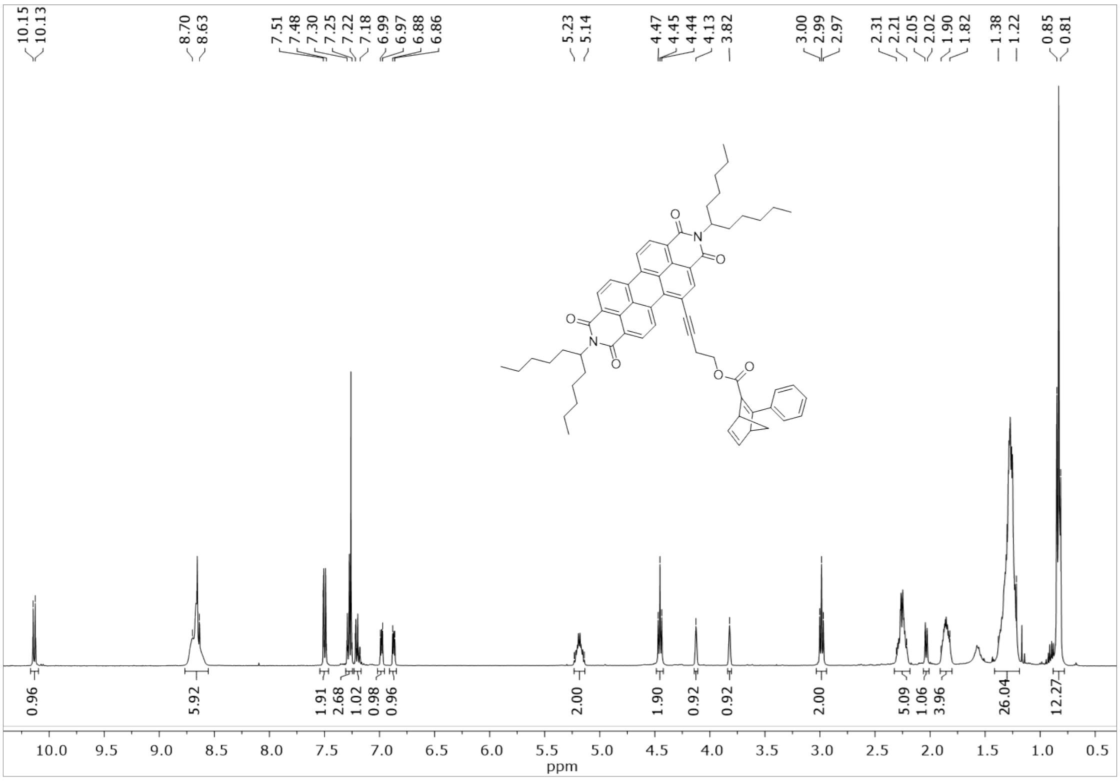

Figure S16. ^{1}H NMR spectrum (400 MHz, rt, $CDCl_3$) of **7**-NBD.

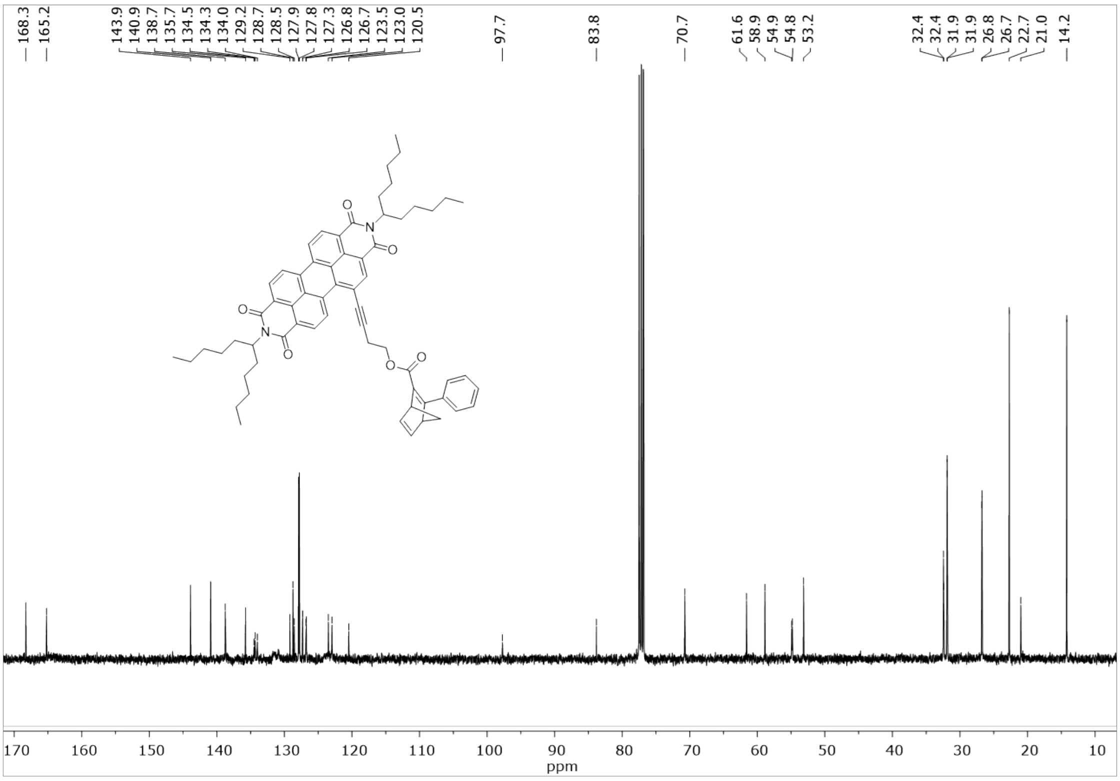

Figure S17. ^{13}C NMR spectrum (101 MHz, rt, $CDCl_3$) of **7**-NBD.

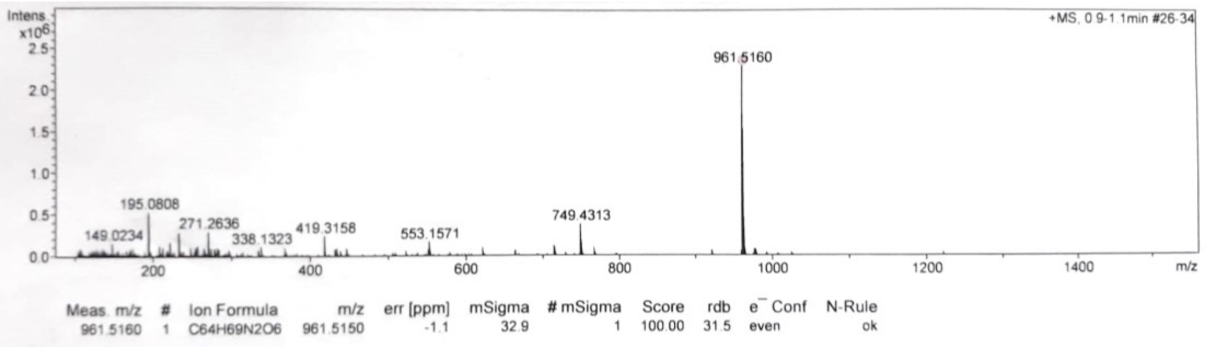

Figure S18. Mass spectrum of **7**-NBD in DCM.

Experimental Procedures

General Methods

If not mentioned otherwise, all reagents and chemicals were bought from Sigma-Aldrich or Alfa Aesar and used without purification.

For TLC, silica gel 60 F254 (Merck) was used and visualized by a UV lamp (λ = 254 and 365 nm), if necessary.

For column chromatography, silica gel 60 M (deactivated, 0.04 – 0.063 mm) was used (Macherey-Nagel).

For flash chromatography, a Biotage Selekt device was used.

All NMR measurements were performed on a Bruker Avance 300 or an Avance 400.

Steady state absorption spectra were obtained using a PerkinElmer Lambda 2 dual beam absorption spectrometer or a Shimadzu UV1900i absorption spectrometer with scan rates of 480 nm/min.

Steady state emission spectra were recorded and irradiation experiments were conducted using a Horiba Jobin Yvon FluoroMax-3 emission spectrometer or an Edinburgh FS5 Emission spectrometer with 5 nm spectral bandwidth and 0.1s integration time.
For irradiation experiments, samples were purged with Ar for 30 min.

Fluorescence Quantum Yields were determined using an Edinburgh FS5 emission spectrometer mounted with the SC-30 integration sphere module. Samples were contained in 10 x 10 mm quartz cuvettes and purged with Ar for 30 min.

Time-correlated single-photon counting was carried out using a Horiba Jobin Yvon FluoroLog-3 and Edinburgh FS5 spectrofluorometer setup. A Picoquant Vis/UV was used as excitation light source with a Hamamatsu MCP photomultiplier (R33809U-50 and R928P) as a detector. Fit and lifetime analysis were performed with the TCSPC software and FLUORACLE software of the instruments or using Origin software. Samples were contained in 10×10 mm quartz cuvettes and purged with argon for 30 min.

Femtosecond and nanosecond transient absorption studies were performed with the HELIOS (0 to 5500 ps) or EOS system (1 ns to 350 µs) from Ultrafast Systems. The laser source was a Clark MXR CPA2110 and CPA2101 Ti:sapphire amplifier with a pulsed 1 kHz output centered at 775 nm with 150 fs pulse width. Pump pulses at 532 and 640 nm were generated with a non-collinear optical parametric amplifier (NOPA; Clark-MXR). Excitation pulses at 320 nm were achieved via frequency doubling of the 640 nm pulses. The repetition rate of the pump pulses was set to 525 Hz for fsTA (by means of a chopper) and to 1050 Hz for nsTA. The bandwidth of the excitation pulse is < 15 nm. The white-light for the femtosecond experiments was generated by focusing the 775 nm fundamental output into a 2 mm (vis: 420 –760 nm) or 1 cm (NIR: 800 –1500 nm) Sapphire disk. In the case of nanosecond experiments, the white-light (370 –1600 nm) was generated by a supercontinuum laser source with 2 kHz repetition rate and a pulse width of approximately 1 ns. Samples were contained in 2 x 10 mm quartz cuvettes and purged with argon for 15 min. Global Target Analysis of transient absorption data was performed with the GloTarAn software[3] and includes a chirp correction.

NMR Photoswitching Experiments

Sample preparation

Specific amounts of **5**-NBD and **7**-NBD were dissolved in different deuterated solvents (benzene-d_6, toluene-d_8, $CDCl_3$ or THF-d_8) to obtain solutions with a concentration of 7.93 mmol/L. A volume of 0.45 mL of each solution was transferred to an NMR tube and flushed with argon. Subsequently, each sample was placed in a custom-build photoreactor which contains different LEDs.

LEDs

• 310 nm LED: Imax 600 mA, power 20 mW, Voltage 5.5 V
• 475 nm LED: Imax 350 mA, power 3 W, Voltage 3.2 V

Measurements

The samples were irradiated inside the photoreactor, and NMR measurements were performed after specific times to follow the reaction processes. The exact times and conversion rates of the measurements are presented in the tables below.

Data analysis procedure

The analysis of 5-NBD in THF-d8 will now be explained in detail, representative for all the other NMR spectra analysis, showing the general data analysis procedure.
Before the sample was irradiated, a NMR start measurement was performed (see Figure S14, bottom).For every measured NMR spectrum an automatic phase correction and a baseline correction (polynomial order 5) was performed. The integral of the signal at around 5.20 ppm, which is characteristic for a PDI (see Figure S14, green box), is set to 2 and used as integration reference signal, as this signal is not changing during the irradiation experiments. One olefinic proton of the NBD (see Figure S14, marked in blue) is integrated and used to determine the amount of converted NBD. In the start measurement spectrum, the NBD signal is integrated to 0.97 which is set to 100% of NBD. After irradiating the sample for 210 minutes at 310 nm, the integration of the NBD peak has reduced to 0.14 (see Figure S14, center) which indicates a remaining amount of 14% of NBD (rounded, 0.97/0.14 = 14.43%). Subsequent irradiation of the same sample for 30 minutes at 475 nm leads to an increase of the NBD signal (see Figure S14, top) to an integration value of 0.63, which dedicates an amount of 65 % of NBD (rounded, 0.63/0.97 = 64.94%). As already mentioned in the paper, the conversion in THF-d_8 is not reversible, as only an amount of 65% of the originally used NBD was measured after the experiment.

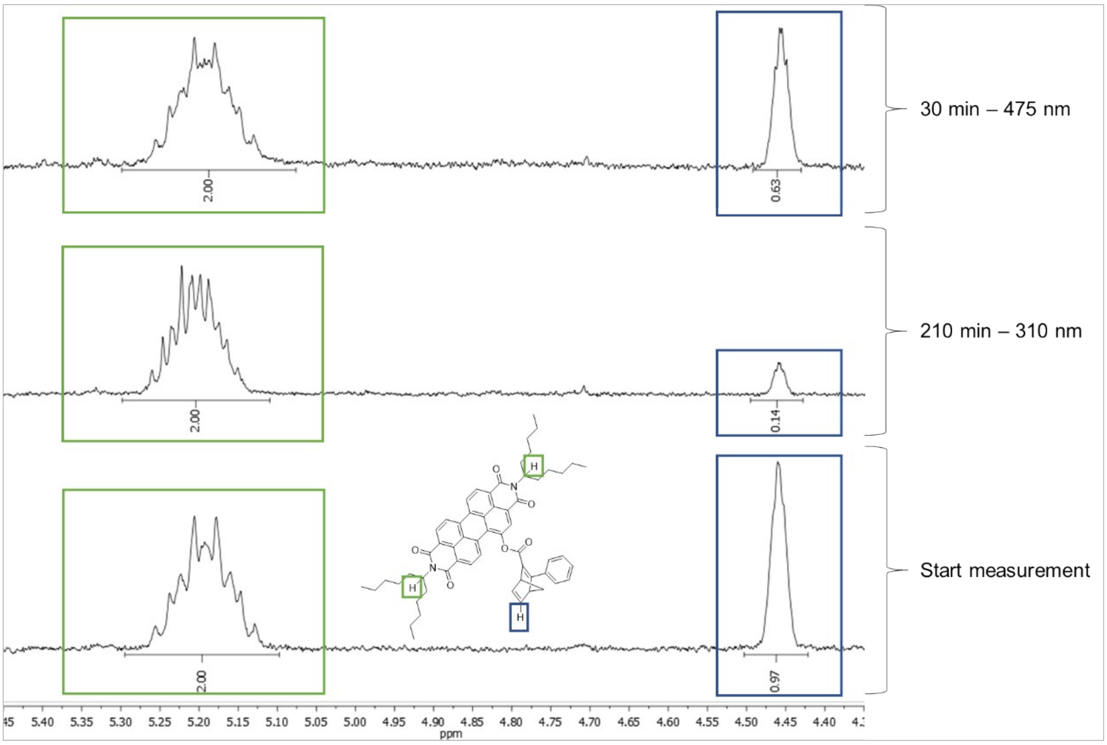

Figure S19. Bottom: ^1H NMR spectrum of **5**-NBD in THF-d_8 before irradiation. Middle: ^1H NMR spectrum of **5**-NBD in THF-d_8 after irradiation at 310 nm for 210 minutes. Top: ^1H NMR spectrum of **5**-NBD in THF-d_8 after irradiation at 310 nm for 210 minutes, followed by irradiation at 475 nm for 30 minutes.

Results and Discussion

Computational Details

At the beginning, a benchmark test of the isomerization enthalpies of the pure NBD-QC rearrangement in gas phase was performed, testing five unique functionals (B3LYP[4], CAM-B3LYP[5], BMK[6], wB97xD[7], M06-2X[8]) from different density functional families. The experimentally determined and commonly cited value of 89 kJ/mol was used as the reference value.[9] Furthermore, three basis sets from the Ahlrichs family (def2SVP, def2TZVP, def2QZVP)[10,11] were used for the benchmark study. As seen in Table S1 and Figure S15, only the isomerization enthalpy determined with CAM-B3LYP agrees well with the experimental value. Furthermore, all methods converged very well with the def2TZVP basis set and further increasing the basis set size did not significantly improve the results. For this reason, the molecules studied were calculated at the CAM-B3LYP/def2TZVP level. Since dispersion interactions play an important role in the accuracy of the calculation, Grimme's dispersion model with Becke-Johnson damping (GD3BJ) was included.[12] Further, corrections for zero-point energies were incorporated for the determination of isomerization enthalpies.

Table S1. Isomerization enthalpies of the NBD-QC reorganization calculated with five unique functionals and three Ahlrichs basis sets.

Functional	ΔE def2SVP [kJ/mol]	ΔE def2TZVP [kJ/mol]	ΔE def2QZVP [kJ/mol]
B3LYP	82.15	101.16	101.79
CAM-B3LYP	64.99	85.18	85.86
BMK	66.12	69.91	67.66
wB97xD	52.21	68.98	69.37
M06-2X	45.31	59.59	60.38

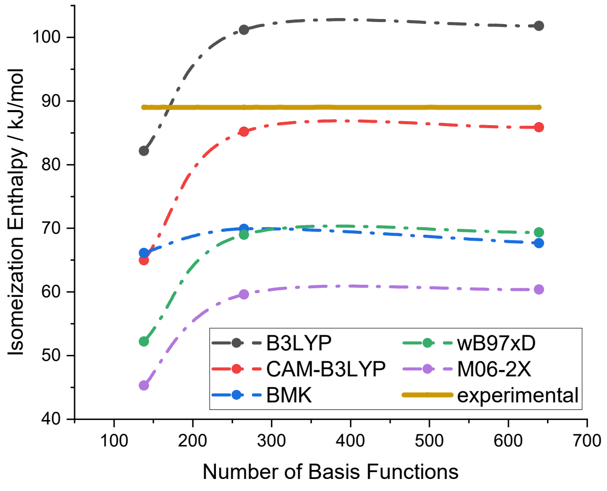

Figure S20. Isomerization enthalpies of the NBD-QC reorganization as a function of the size of the used basis sets.

Table S2. Ground state energies and isomerization enthalpies of **5**-NBD, **5**-QC, **7**-NBD, and **7**-QC.

	E(HF) [Hartree]	E(HF) [kcal/mol]	E(ZPE) [kcal/mol]	E [kcal/mol]
**5**-NBD	-2099.48046	-1317423.99	347.78	-1317076.21
**7**-NBD	-2254.24510	-1414538.80	391.23	-1414147.58
**5**-QC	-2099.44285	-1317400.39	347.22	-1317053.17
**7**-QC	-2254.20868	-1414515.94	390.68	-1414125.27

	ΔE [kcal/mol]	ΔE [kJ/mol]
**5**-NBD → **5**-QC	23.04	96.42
**7**-NBD → **7**-QC	22.31	93.33

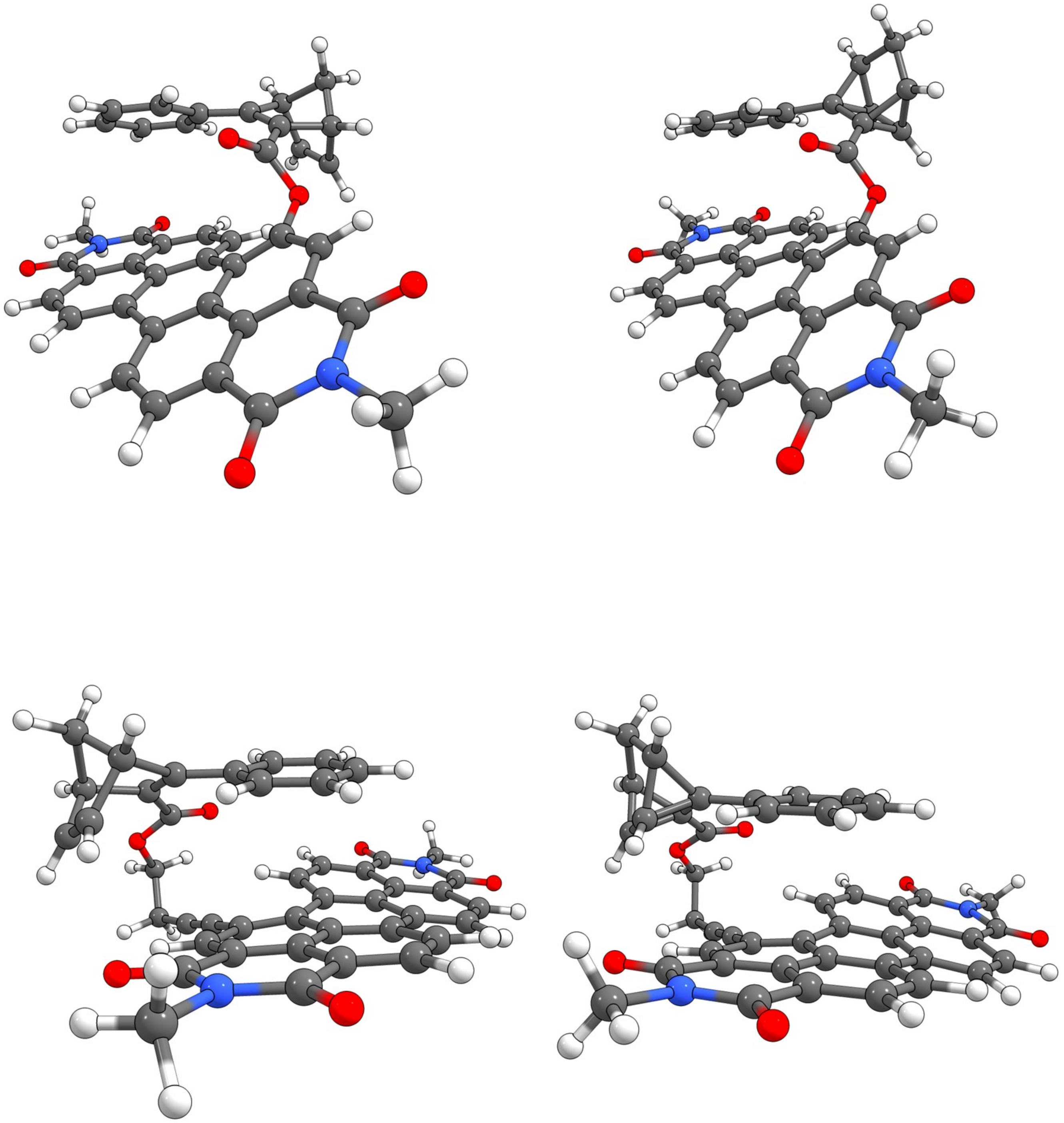

Figure S21. CAM-B3LYP/def2TZVP optimized structures of **5**-NBD (top left), **5**-QC (top right), **7**-NBD (bottom left) and **7**-QC (bottom right).

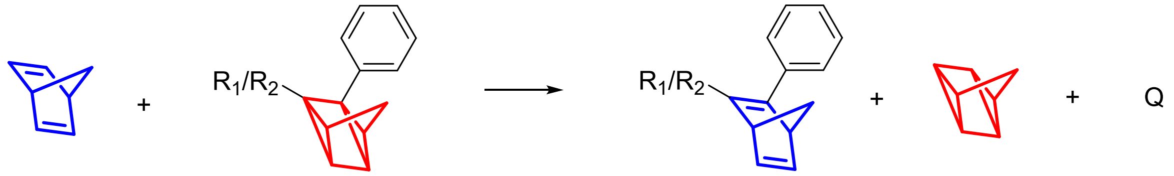

R_1:	-712748 kJ/mol	-5513541 kJ/mol	-5513657 kJ/mol	-712647 kJ/mol	**15 kJ/mol**
R_2:		-5919963 kJ/mol	-5920075 kJ/mol		**11 kJ/mol**

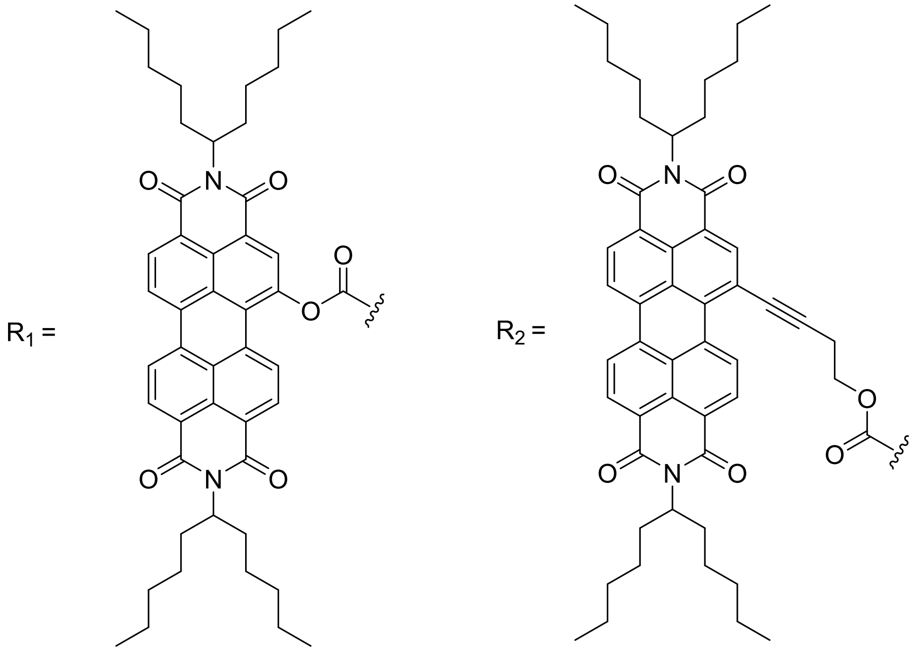

Scheme S1. Isodesmic reaction scheme of **5**-QC to **5**-NBD and **7**-QC to **7**-NBD with the corresponding formation enthalpies and the resulting heat of the isodesmic reaction (Q).

Additional Spectra

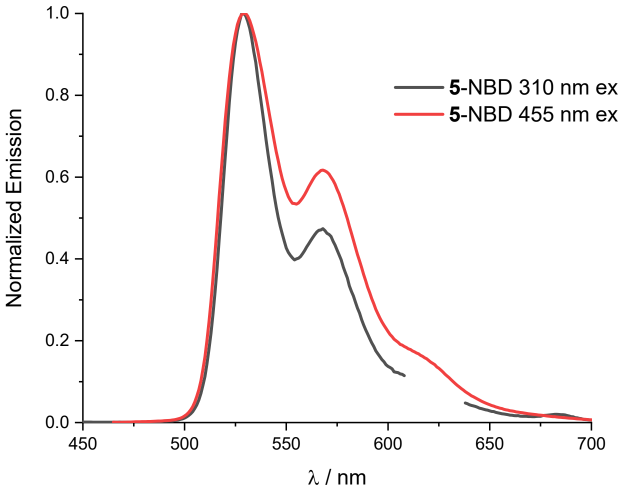

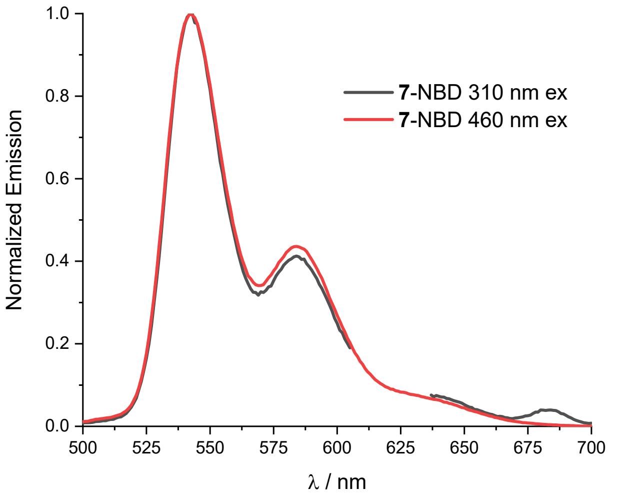

Figure S22. Top: Fluorescence spectra of **5**-NBD in THF obtained upon 310 nm photoexcitation (black) and 455 nm photoexcitation (red). Bottom: Fluorescence spectra of **7**-NBD in THF upon 310 m photoexcitation (black) and 460 nm excitation (red).

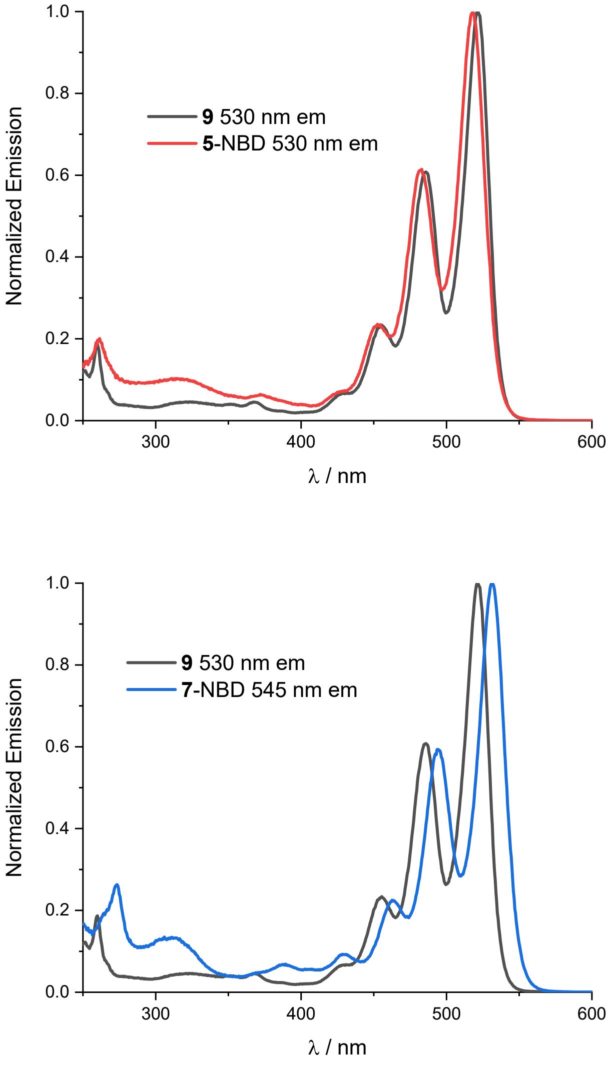

Figure S23. Top: Excitation spectra of **9** (black) and **5**-NBD (red) recorded at the 530 nm fluorescence in THF. Bottom: Excitation spectra of **9** (black) and **7**-NBD (blue) recorded at the 530 and 545 nm fluorescence, respectively, in THF

Table S3. TCSPC lifetimes determined at the 560 nm fluorescence of **9, 5**-NBD, **5**-QC, **7**-NBD, and **7**-QC in THF.

	Fluorescence lifetime at 355 nm excitation [ns]	Fluorescence lifetime at 532 nm excitation [ns]
**9**	4.72	4.22
**5**-NBD	4.48	---
**5**-QC	4.71	4.22
**7**-NBD	4.88	---
**7**-QC	5.03	4.41

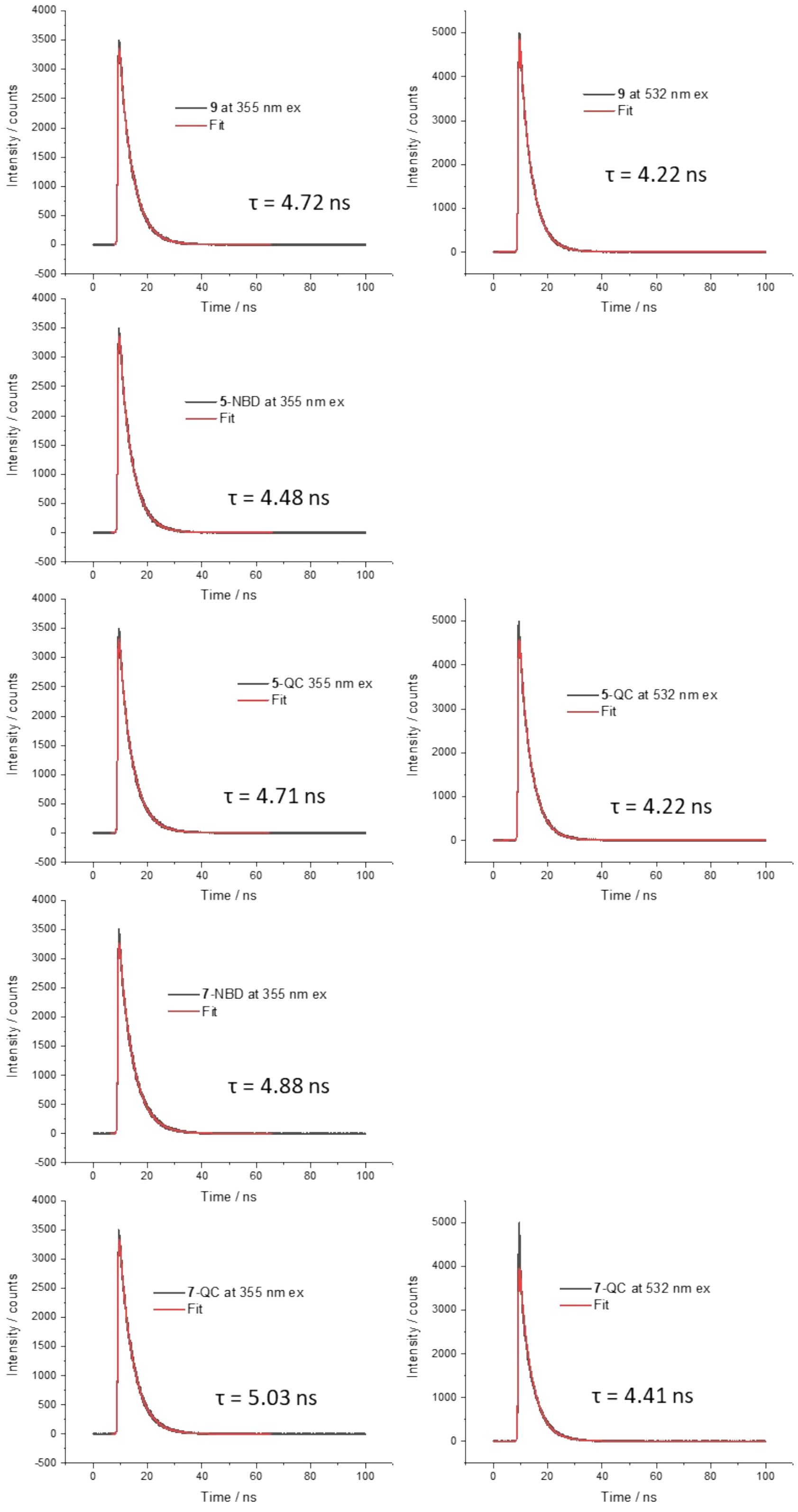

Figure S24. TCSPC decays and lifetimes of **9**, **5**-NBD, **5**-QC, **7**-NBD and **7**-QC at 355 nm (left) and 532 nm (right) excitation.

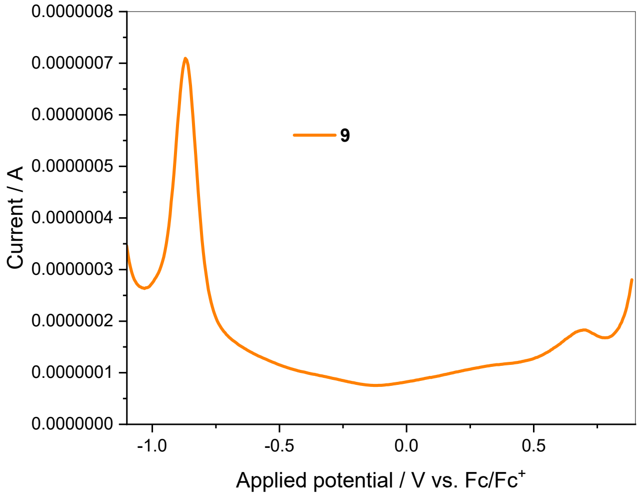

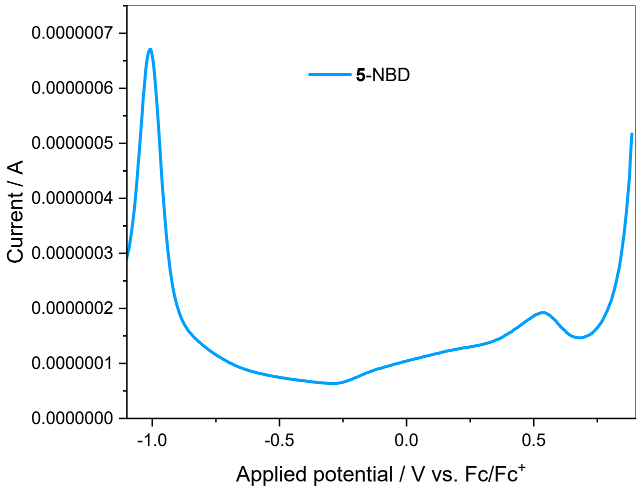

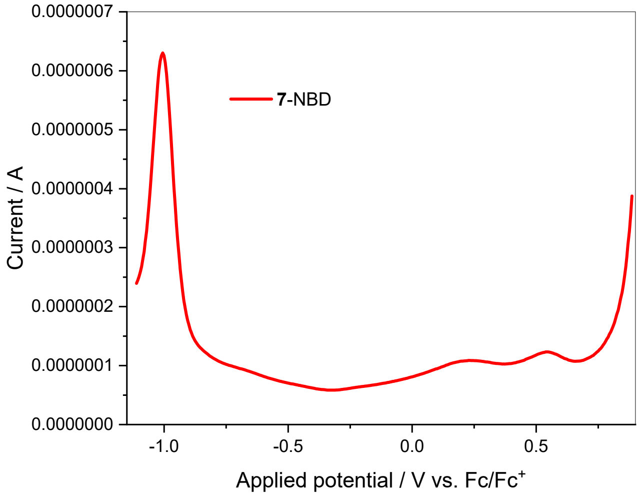

Figure S25. Differential pulse voltammograms of 1 x 10^{-4} M solutions of **9** (top), **5**-NBD (center), and **7**-NBD (bottom) in THF containing 0.1 M $TBAPF_6$.

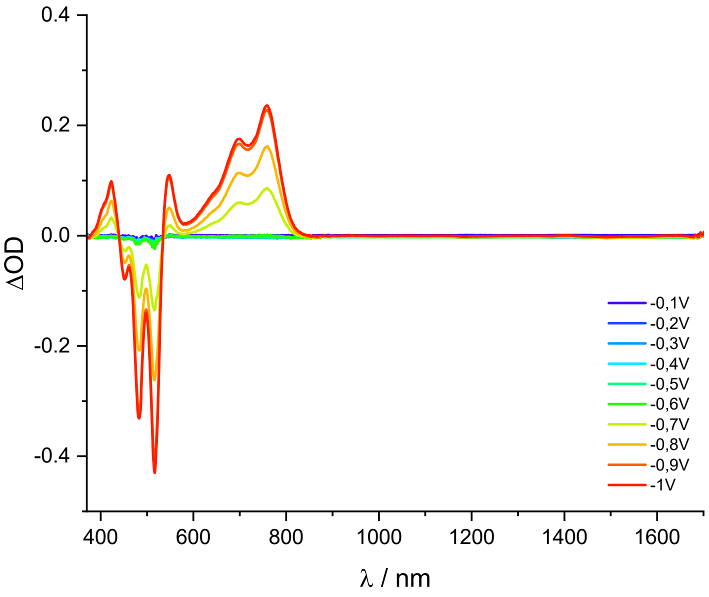

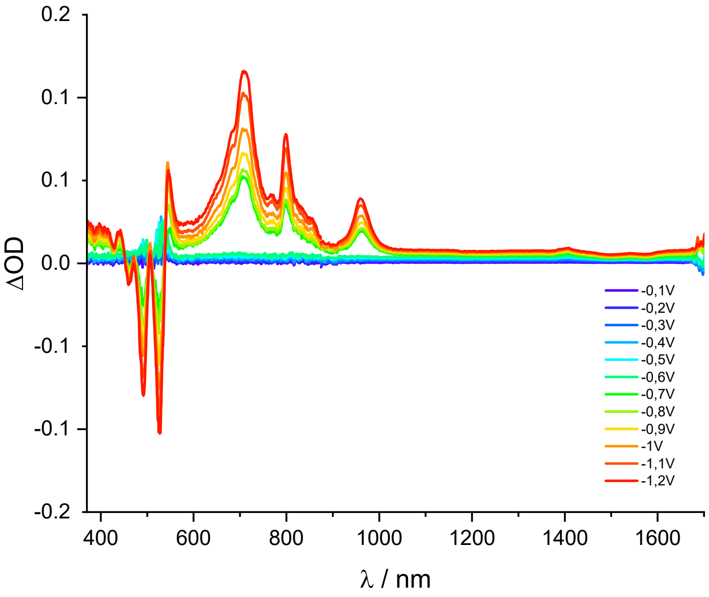

Figure S26. Differential absorption changes obtained upon spectroelectrochemical reduction of 2 x 10^{-5} M **5**-NBD (top) and **7**-NBD (bottom) in THF with 0.1 M $TBAPF_6$ as supporting electrolyte at various potentials from -0.1 to -1.2 V.

Table S4. Percentage of **5**-NBD from ^{1}H NMR after photoirradiation at 310 nm (left) and subsequent photoirradiation at 475 nm (right) in $CDCl_3$.

Time of 310 nm photoirradiation [min]	**5**-NBD [%]	Time of 475 nm photoirradiation [min]	**5**-NBD [%]
0	100	2	91
10	95	4	95
30	88	6	97
60	82	8	96
90	80	10	96

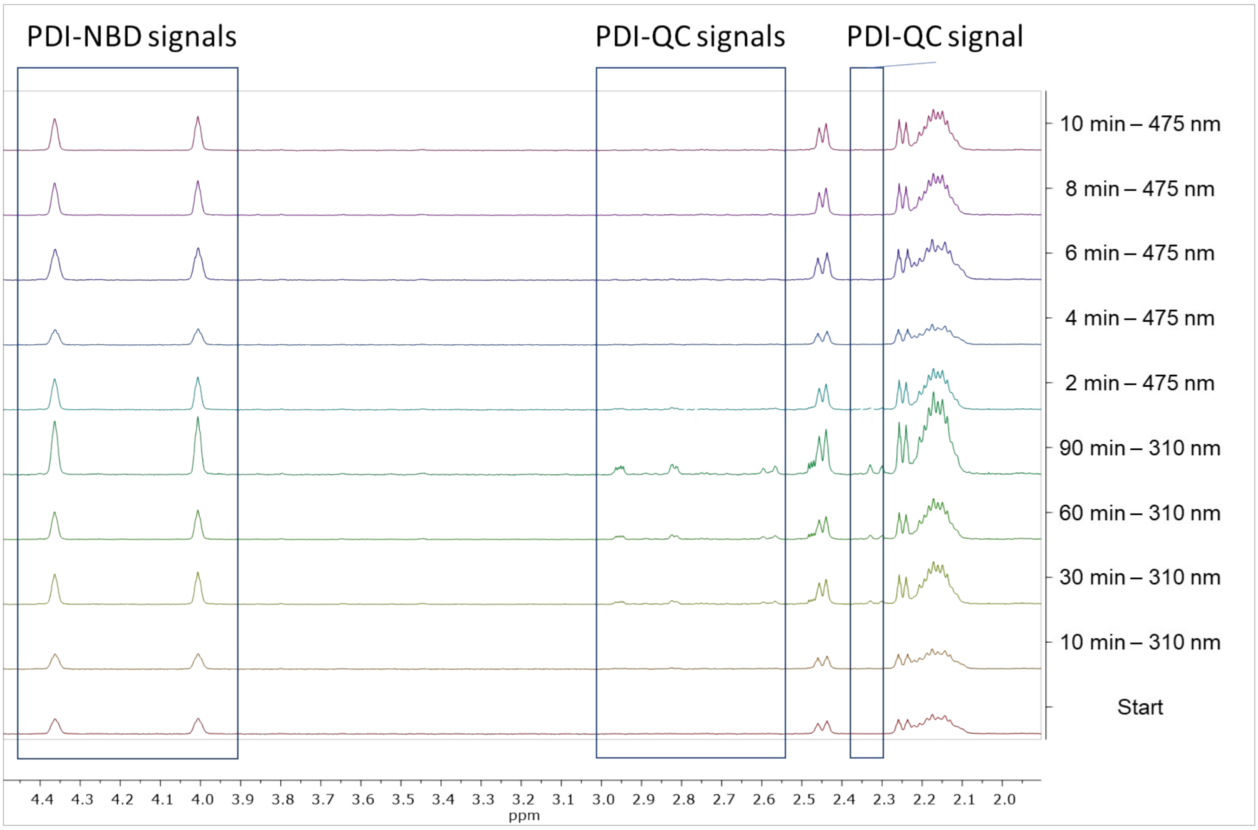

Figure S27. ^{1}H NMR spectra of **5**-NBD in $CDCl_3$ after defined intervals of photoirradiation.

Table S5. Percentage of **5**-NBD from ^{1}H NMR after photoirradiation at 310 nm (left) and subsequent photoirradiation at 475 nm (right) in benzene-d_6.

Time of 310 nm photoirradiation [min]	**5**-NBD [%]	Time of 475 nm photoirradiation [min]	**5**-NBD [%]
0	100	2	86.5
10	97	4	93.5
30	92	6	97.5
60	86	8	98.5
90	82.5	10	99.5
120	80.5	12	100

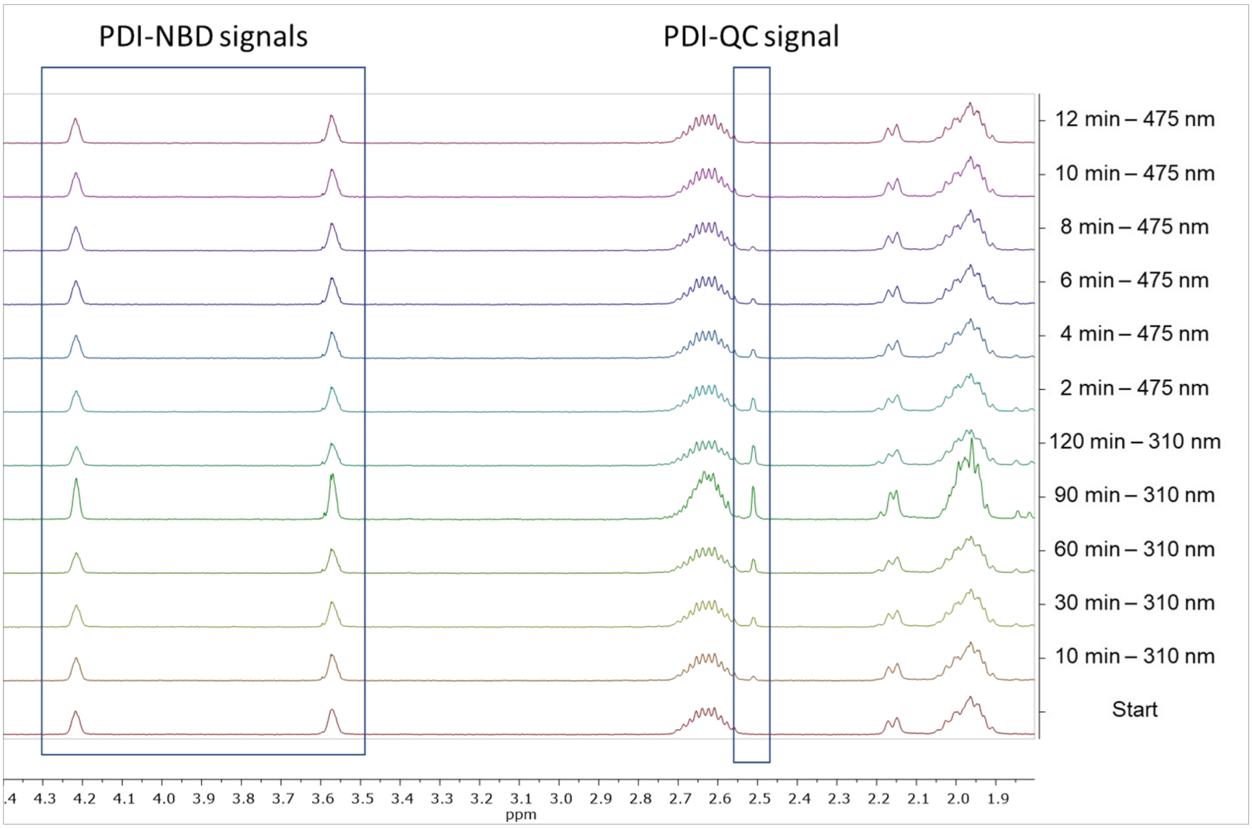

Figure S28. ^{1}H NMR spectra of **5**-NBD in benzene-d_6 after defined intervals of photoirradiation.

Table S6. Percentage of **5**-NBD from ^{1}H NMR after photoirradiation at 310 nm (left) and subsequent photoirradiation at 475 nm (right) in toluene-d_8.

Time of 310 nm photoirradiation [min]	**5**-NBD [%]	Time of 475 nm photoirradiation [min]	**5**-NBD [%]
0	100	2	88
10	96	4	94
30	91	6	98
60	85	8	99
90	80	10	99
120	78		

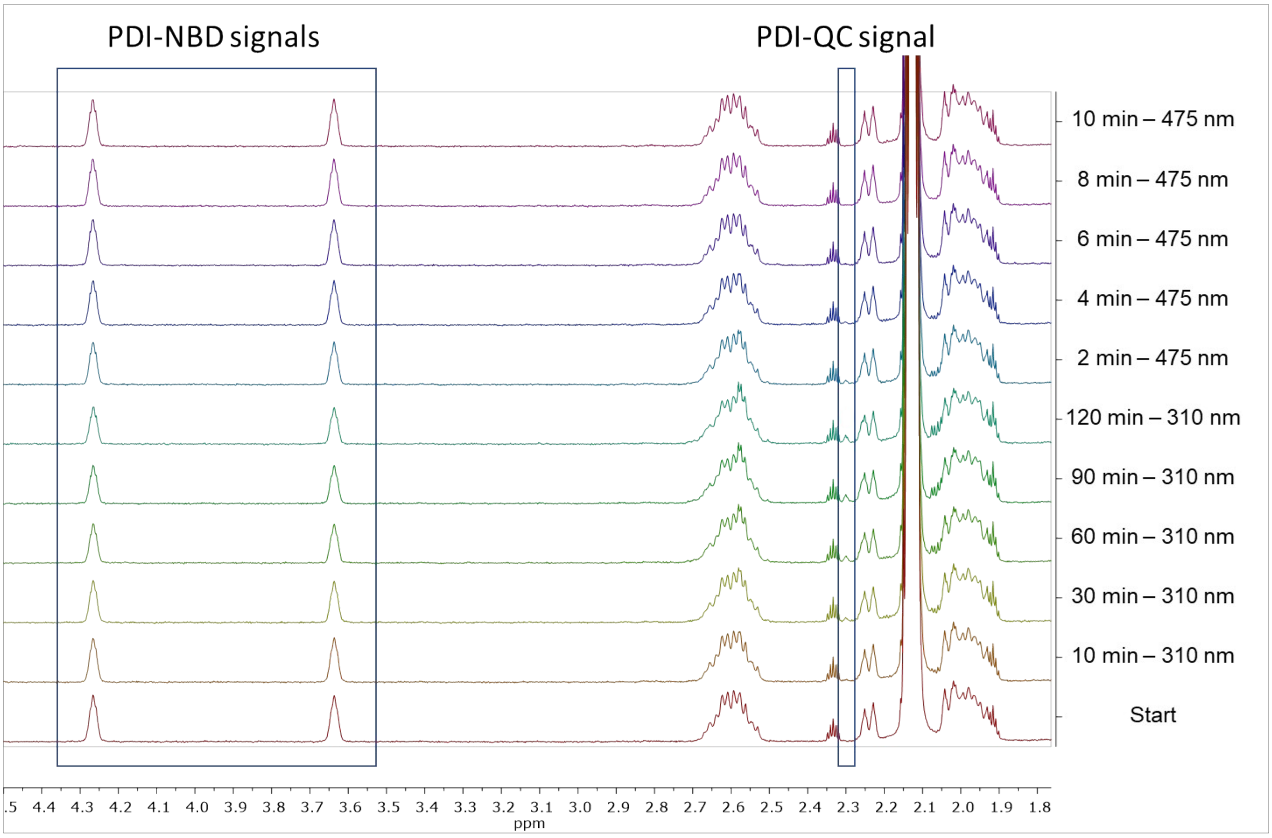

Figure S29. ^{1}H NMR spectra of **5**-NBD in toluene-d_8 after defined intervals of photoirradiation.

Table S7. Percentage of **5**-NBD from ^{1}H NMR after photoirradiation at 310 nm (left) and subsequent photoirradiation at 475 nm (right) in THF-d_8.

Time of 310 nm photoirradiation [min]	**5**-NBD [%]	Time of 475 nm photoirradiation [min]	**5**-NBD [%]
0	100	2	23
10	88	4	28
30	69	8	39
60	46	12	48
90	33	17	55
120	23	23	60
150	19	30	68
180	15		
210	14		

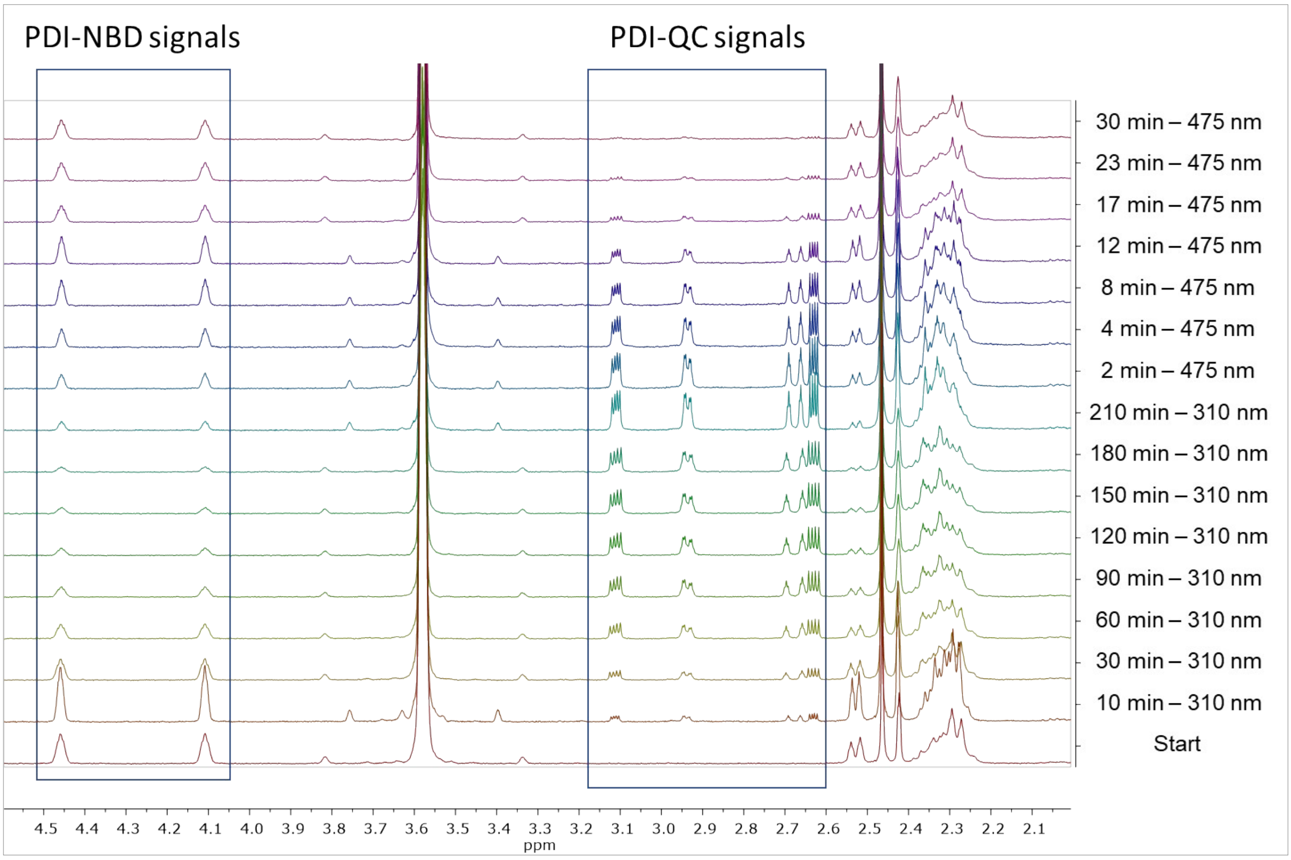

Figure S30. ^{1}H NMR spectra of **5**-NBD in THF-d_8 after defined intervals of photoirradiation.

Table S8. Percentage of **7**-NBD from ^{1}H NMR after irradiation at 310 nm (left) and subsequent photoirradiation at 475 nm (right) in toluene-d_8.

Time of 310 nm photoirradiation [min]	**7**-NBD [%]	Time of 475 nm photoirradiation [min]	**7**-NBD [%]
0	100	2	76
10	92	4	89
30	80	6	91
60	72	8	95
90	67	10	95
120	64	12	96

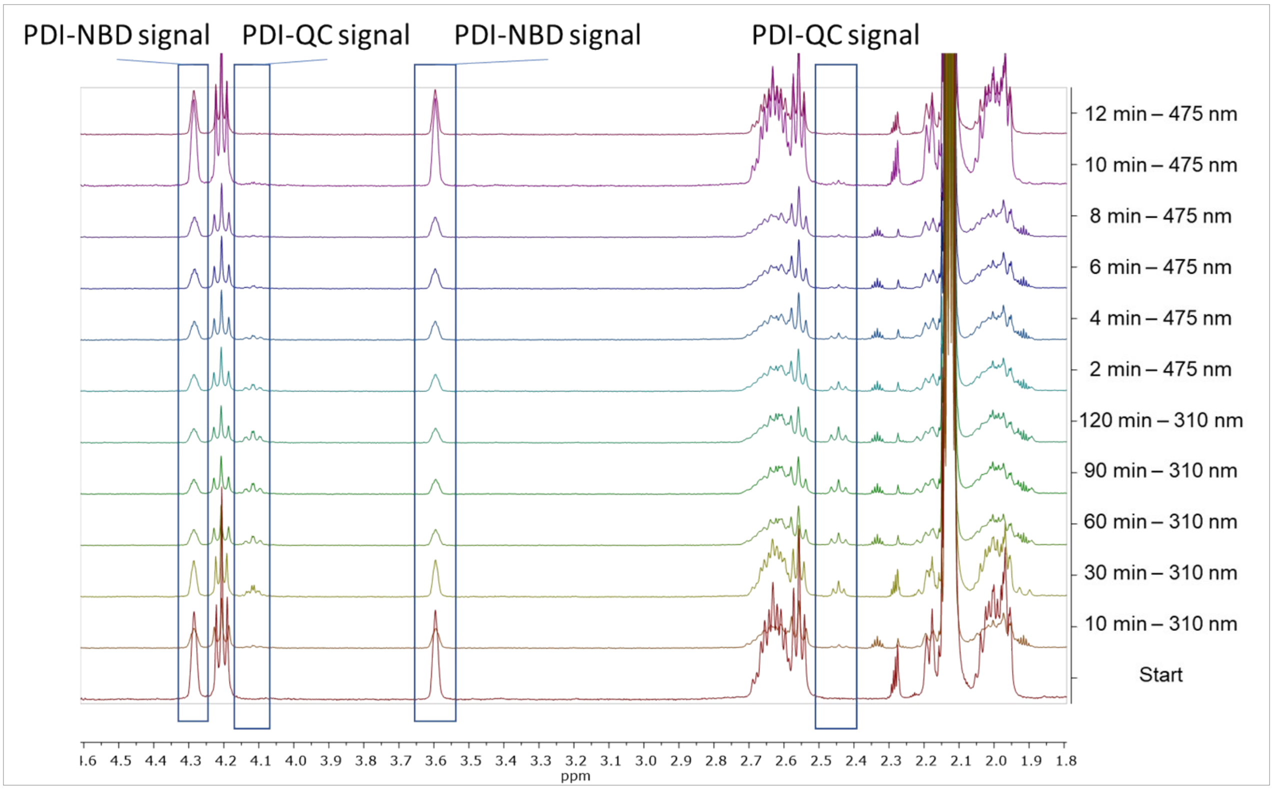

Figure S31. ^{1}H NMR spectra of **7**-NBD in toluene-d_8 after defined intervals of photoirradiation.

Table S9. Percentage of **7**-NBD from ^{1}H NMR after photoirradiation at 310 nm (left) and subsequent photoirradiation at 475 nm (right) in benzene-d_6.

Time of 310 nm photoirradiation [min]	**7**-NBD [%]	Time of 475 nm photoirradiation [min]	**7**-NBD [%]
0	100	2	78
10	96	4	87
30	82	6	93
60	72	8	97
90	66	10	99
120	63	12	100

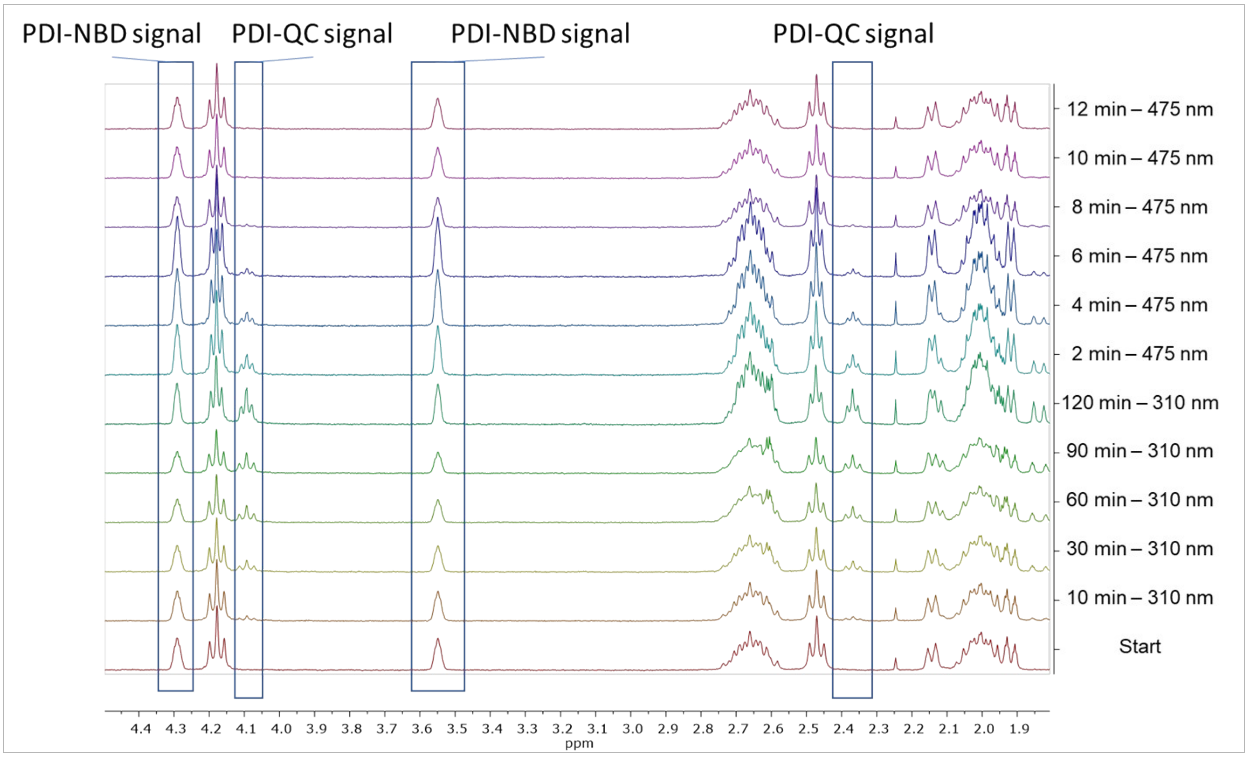

Figure S32. ^{1}H NMR spectra of **7**-NBD in benzene-d_6 after defined intervals of photoirradiation.

Table S10. Percentage of **7**-NBD from ^{1}H NMR after photoirradiation at 310 nm (left) and subsequent photoirradiation at 475 nm (right) in THF-d_8.

Time of 310 nm photoirradiation [min]	**7**-NBD [%]	Time of 475 nm photoirradiation [min]	**7**-NBD [%]
0	100	2	52
10	91	4	65
30	74	8	78
60	58	12	84
90	48	17	88
120	43	23	88
150	38	30	88
180	35		
210	34		

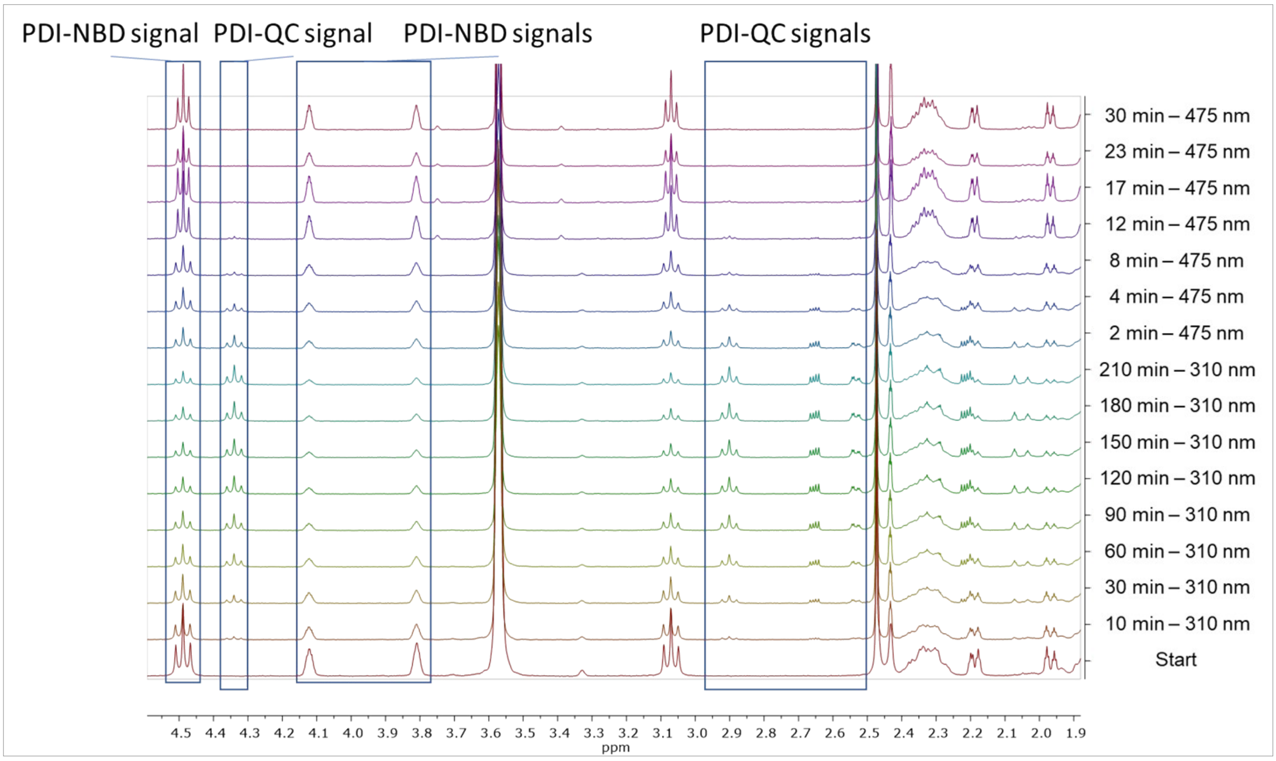

Figure S33. ^{1}H NMR spectra of **7**-NBD in THF-d_8 after defined intervals of photoirradiation.

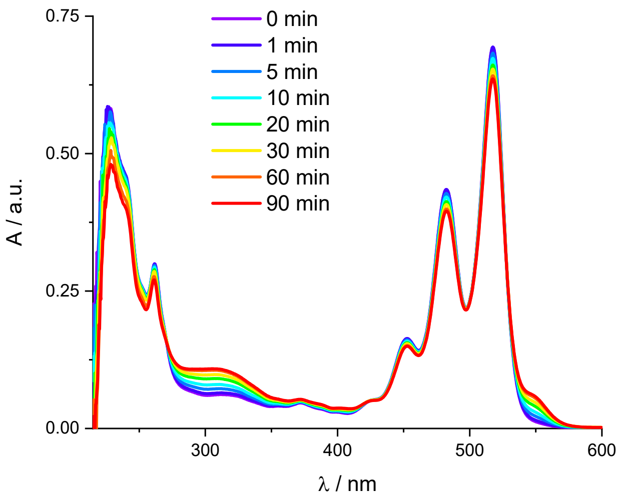

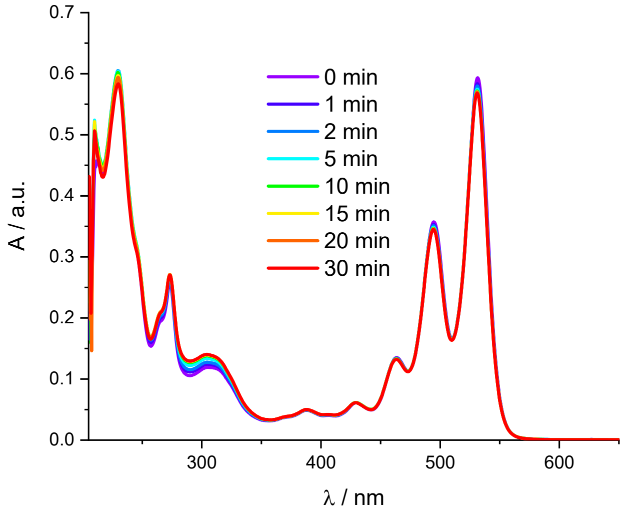

Figure S34. Absorption spectra of 1 x 10^{-5} M **5**-QC (top) and **7**-QC (bottom) in oxygenated THF after 0-90 and 0-30 min of photoirradiation at 532 nm.

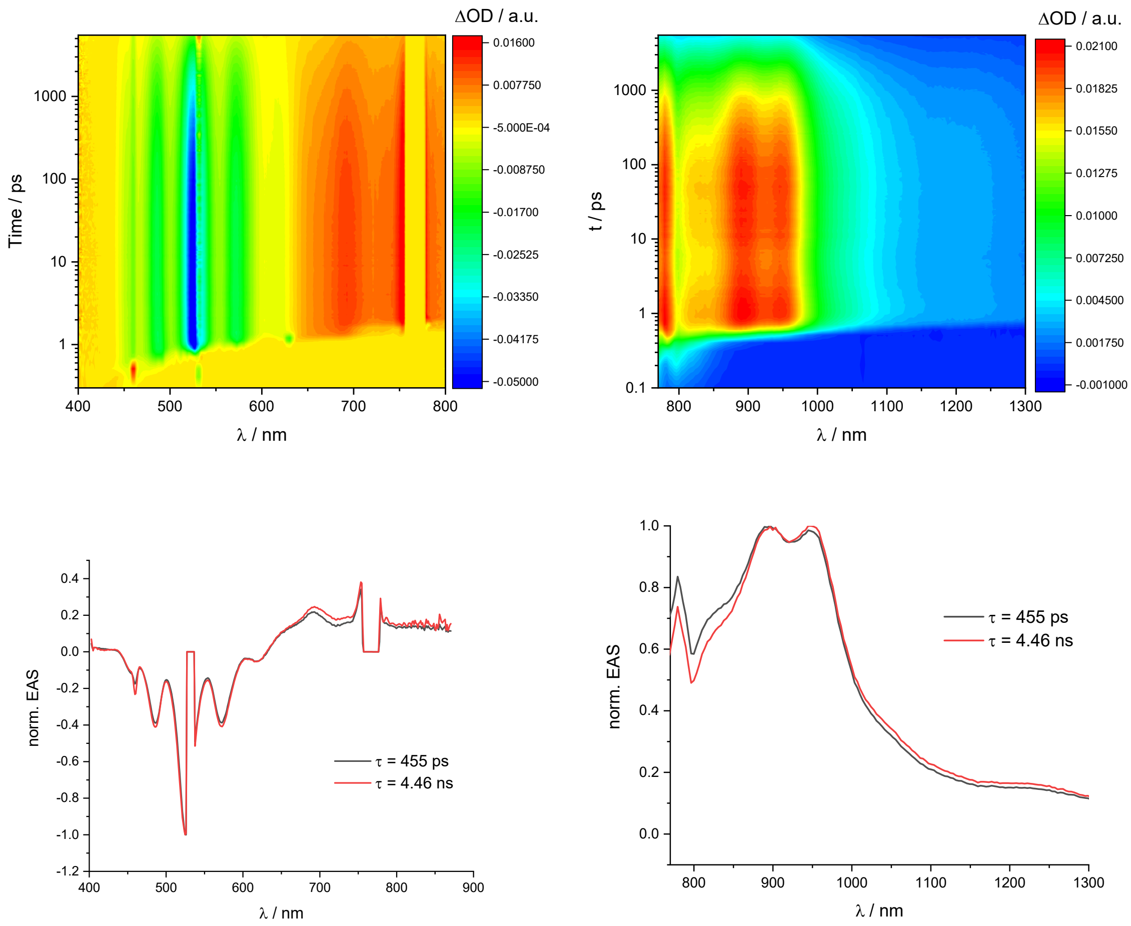

Figure S35. fs-Transient absorption spectroscopy of 1 x 10^{-4} M **9** in THF obtained upon 532 nm photoexcitation with vis detection on the left and NIR detection on the right. Top: 3D plots of the raw data. Bottom: normalized evolution associated spectra obtained by global analysis.

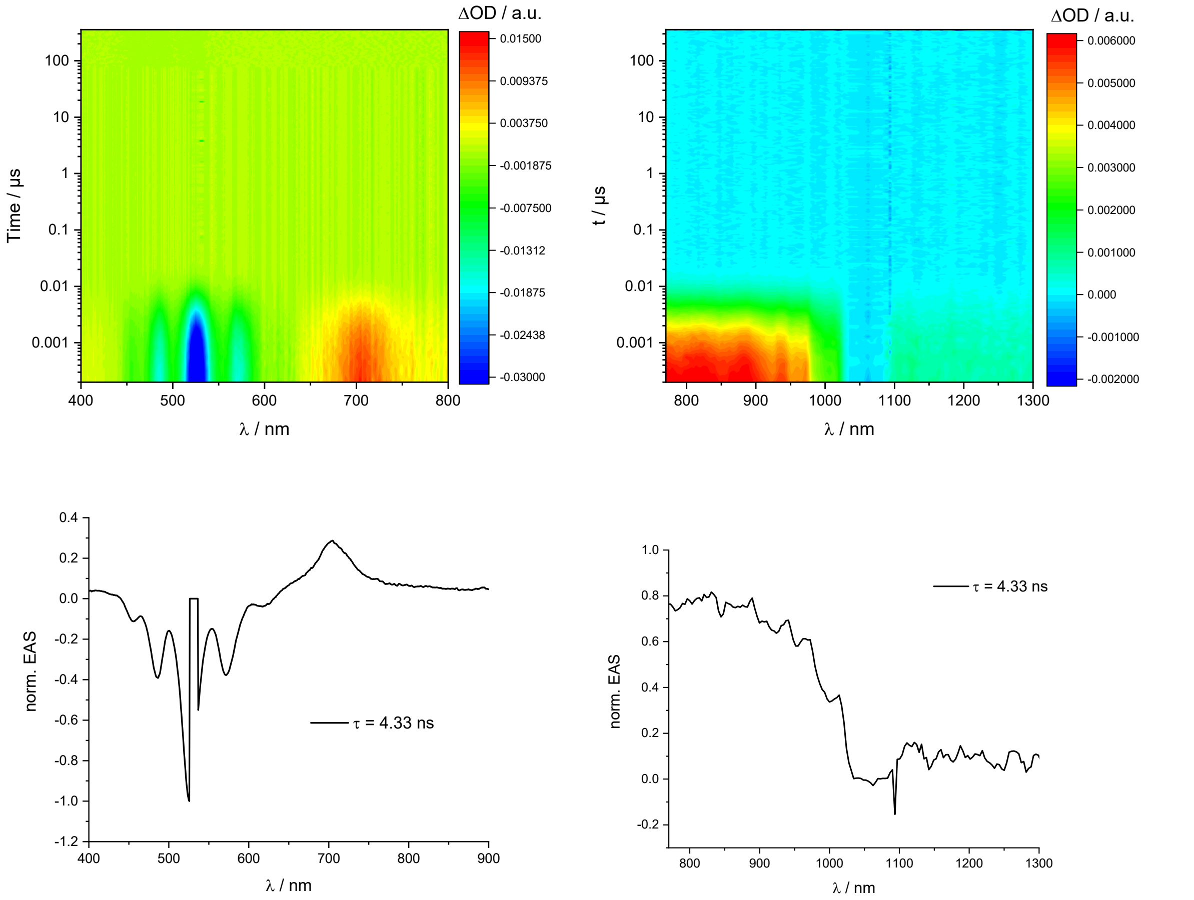

Figure S36. ns-Transient absorption spectroscopy of 1 x 10^{-4} M **9** in THF obtained upon 532 nm photoexcitation with vis detection on the left and NIR detection on the right. Top: 3D plots of the raw data. Bottom: normalized evolution associated spectra obtained by global analysis.

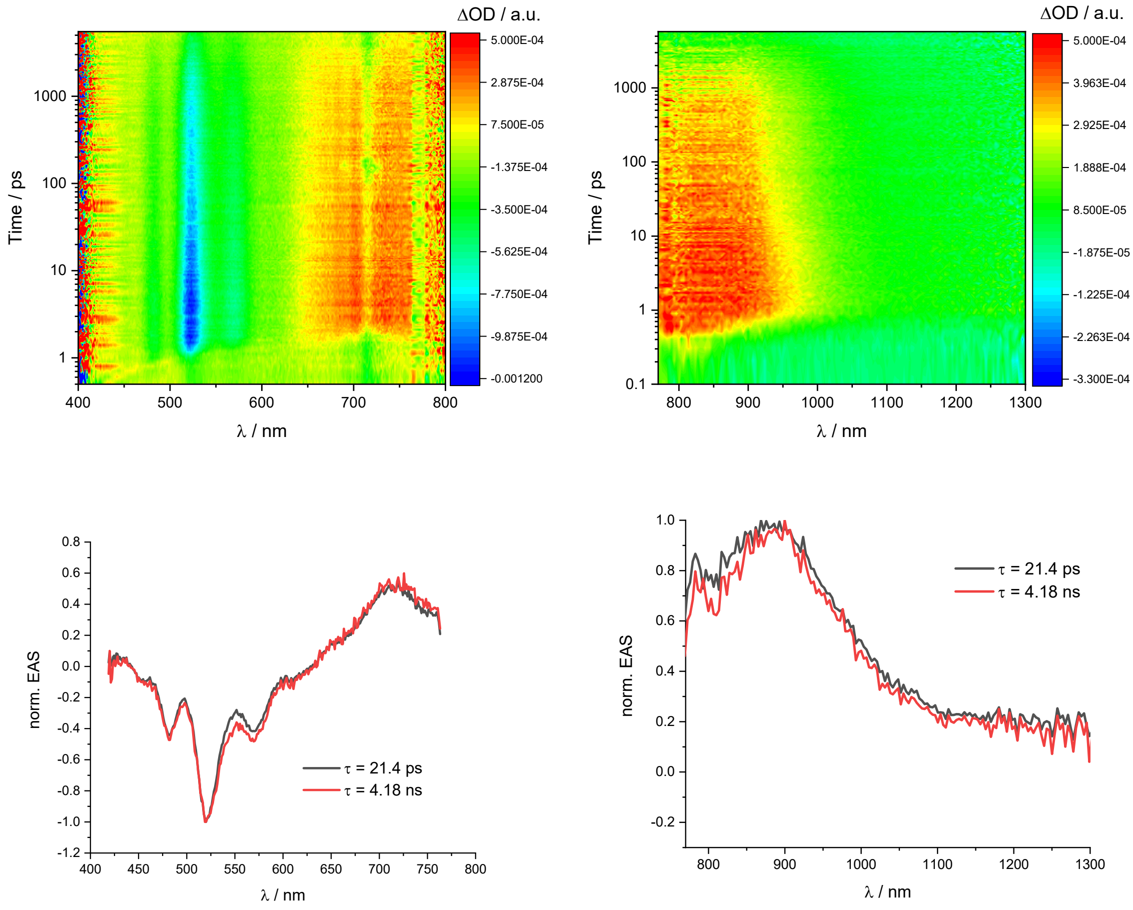

Figure S37. fs-Transient absorption spectroscopy of 1 x 10^{-4} M **5**-NBD in THF obtained upon 310 nm photoexcitation with vis detection on the left and NIR detection on the right. Top: 3D plots of the raw data. Bottom: normalized evolution associated spectra obtained by global analysis.

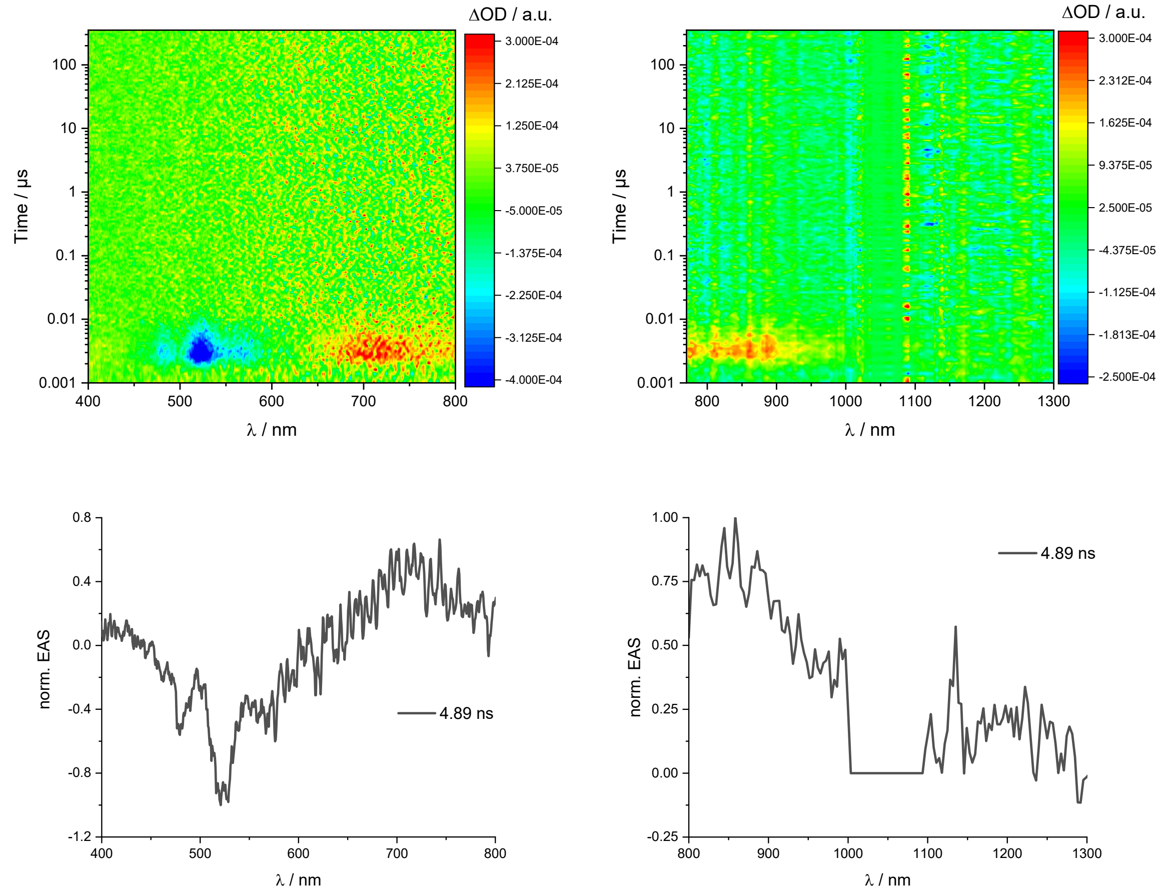

Figure S38. ns-Transient absorption spectroscopy of 1 x 10^{-4} M **5**-NBD in THF obtained upon 310 nm photoexcitation with vis detection on the left and NIR detection on the right. Top: 3D plots of the raw data. Bottom: normalized evolution associated spectra obtained by global analysis.

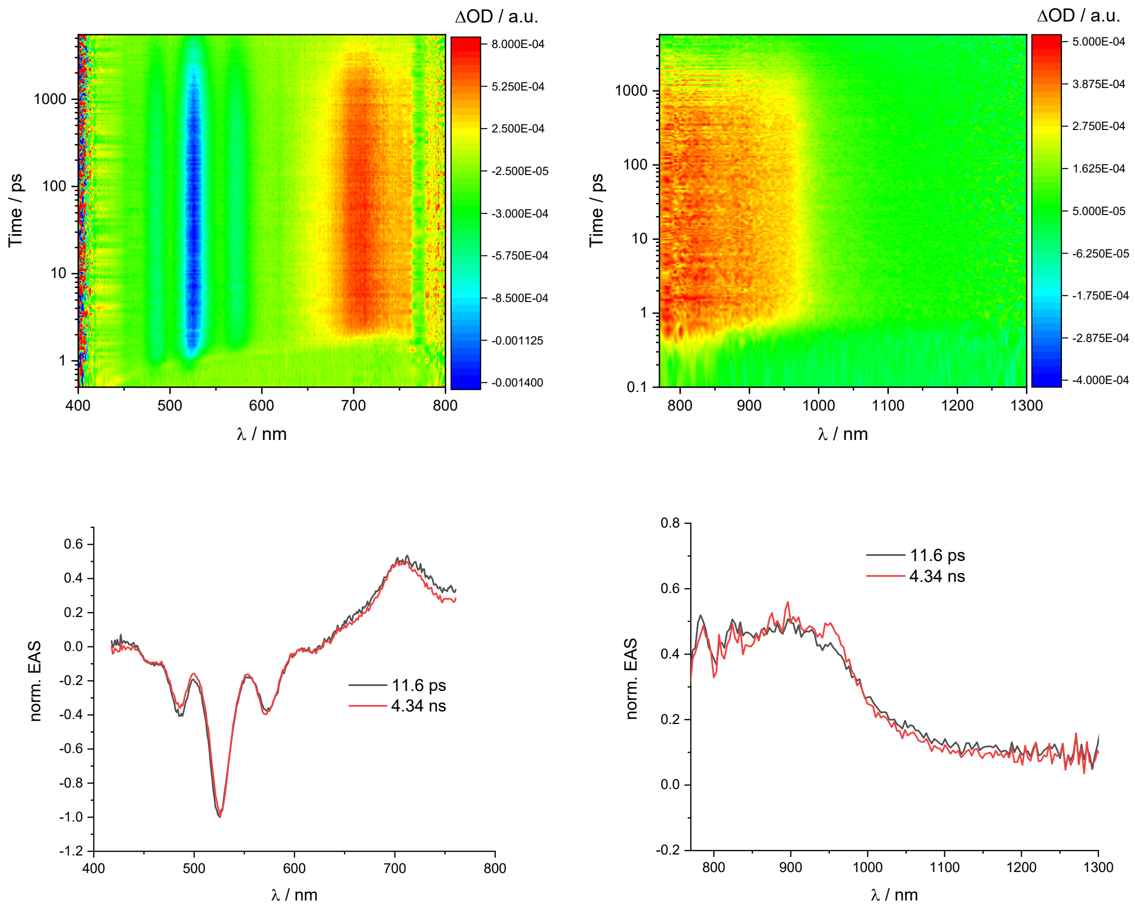

Figure S39. fs-Transient absorption spectroscopy of 1 x 10^{-4} M **9** in THF obtained upon 310 nm photoexcitation with vis detection on the left and NIR detection on the right. Top: 3D plots of the raw data. Bottom: normalized evolution associated spectra obtained by global analysis.

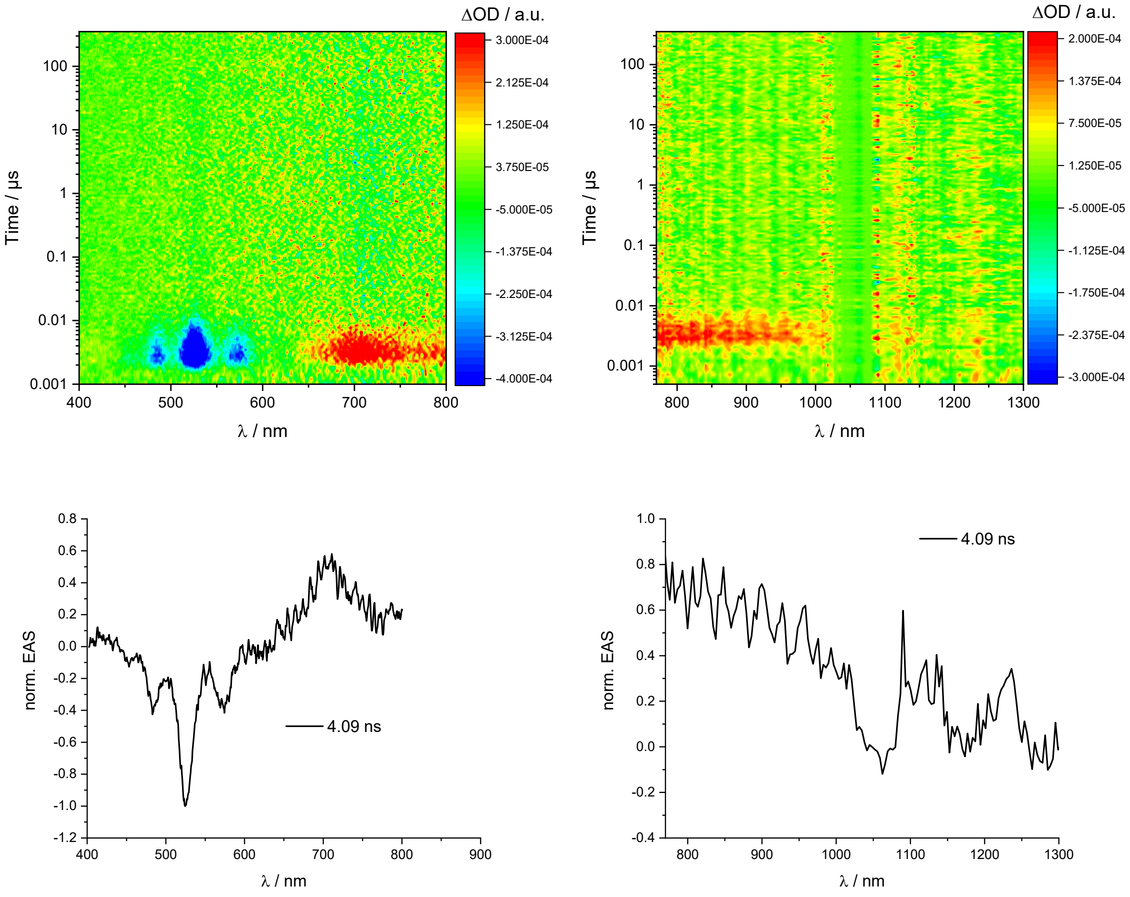

Figure S40. ns-Transient absorption spectroscopy of 1 x 10^{-4} M **9** in THF obtained upon 310 nm photoexcitation with vis detection on the left and NIR detection on the right. Top: 3D plots of the raw data. Bottom: normalized evolution associated spectra obtained by global analysis.

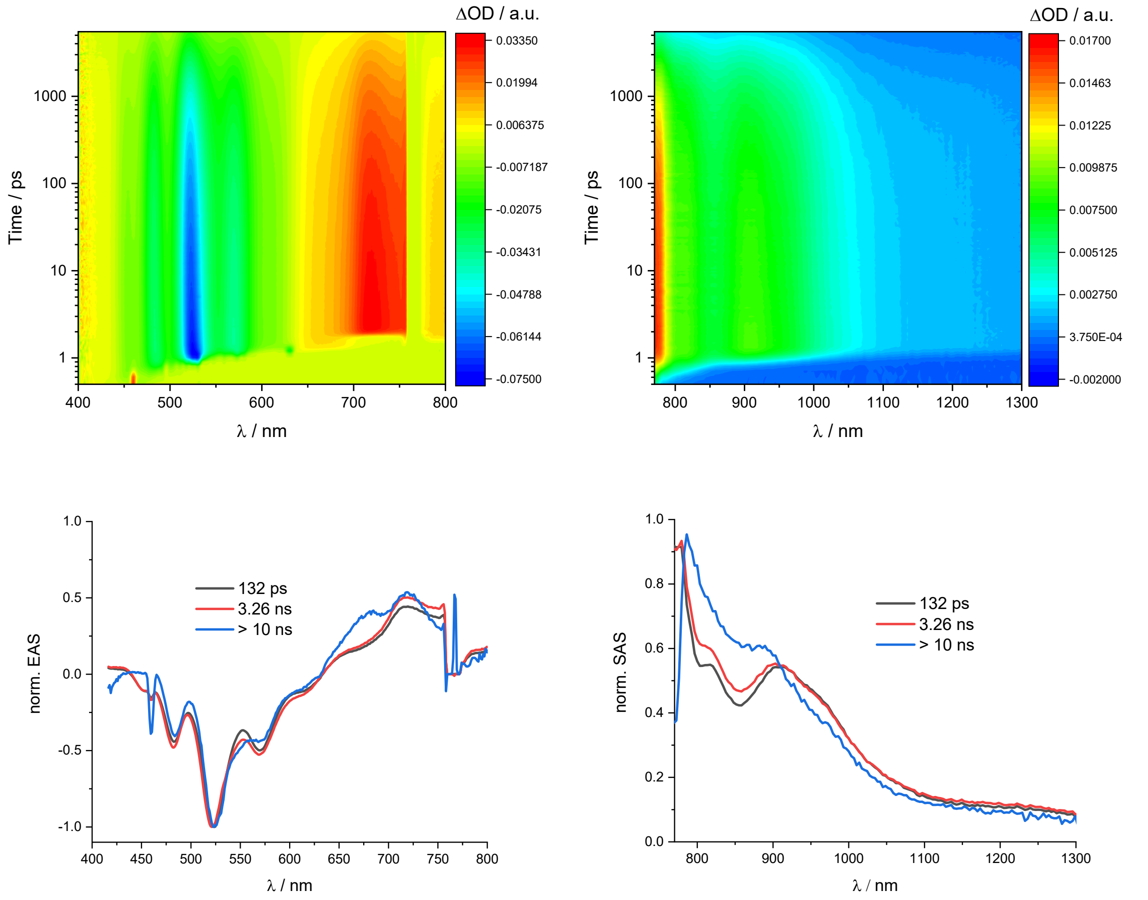

Figure S41. fs-Transient absorption spectroscopy of 1 x 10^{-4} M **5**-QC in THF obtained upon 532 nm photoexcitation with vis detection on the left and NIR detection on the right. Top: 3D plots of the raw data. Bottom: normalized species associated spectra obtained by target analysis.

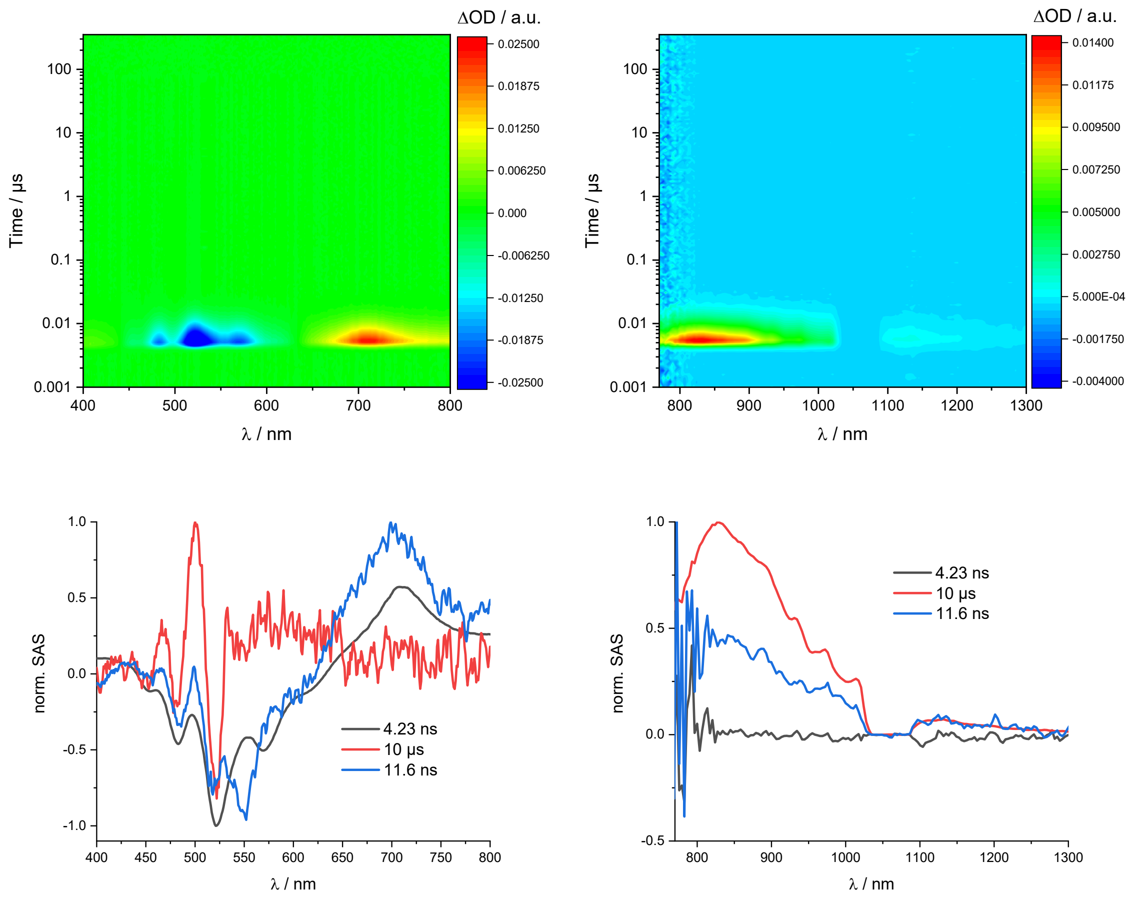

Figure S42. ns-Transient absorption spectroscopy of 1 x 10^{-4} M **5**-QC in THF obtained upon 532 nm photoexcitation with vis detection on the left and NIR detection on the right. Top: 3D plots of the raw data. Bottom: normalized species associated spectra obtained by target analysis.

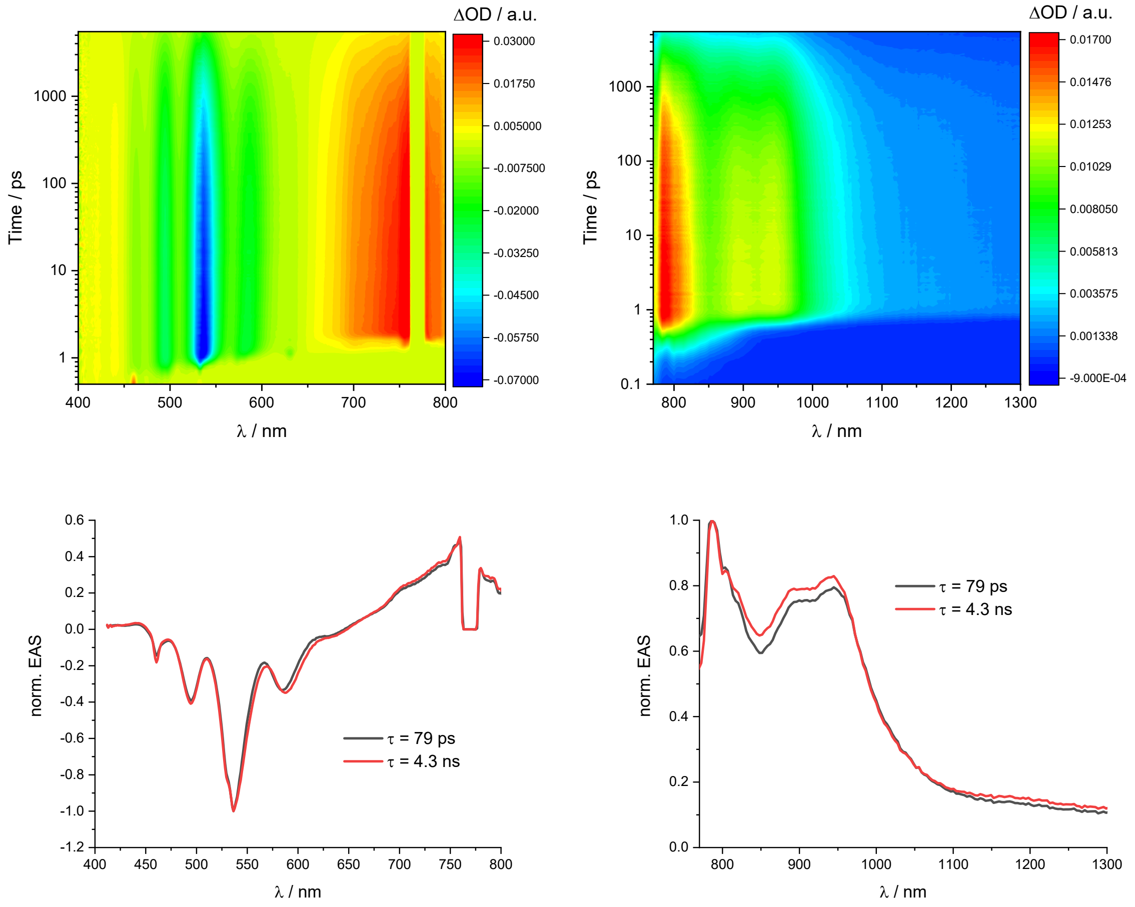

Figure S43. fs-Transient absorption spectroscopy of 1 x 10^{-4} M **7**-QC in THF obtained upon 532 nm photoexcitation with vis detection on the left and NIR detection on the right. Top: 3D plots of the raw data. Bottom: normalized evolution associated spectra obtained by global analysis.

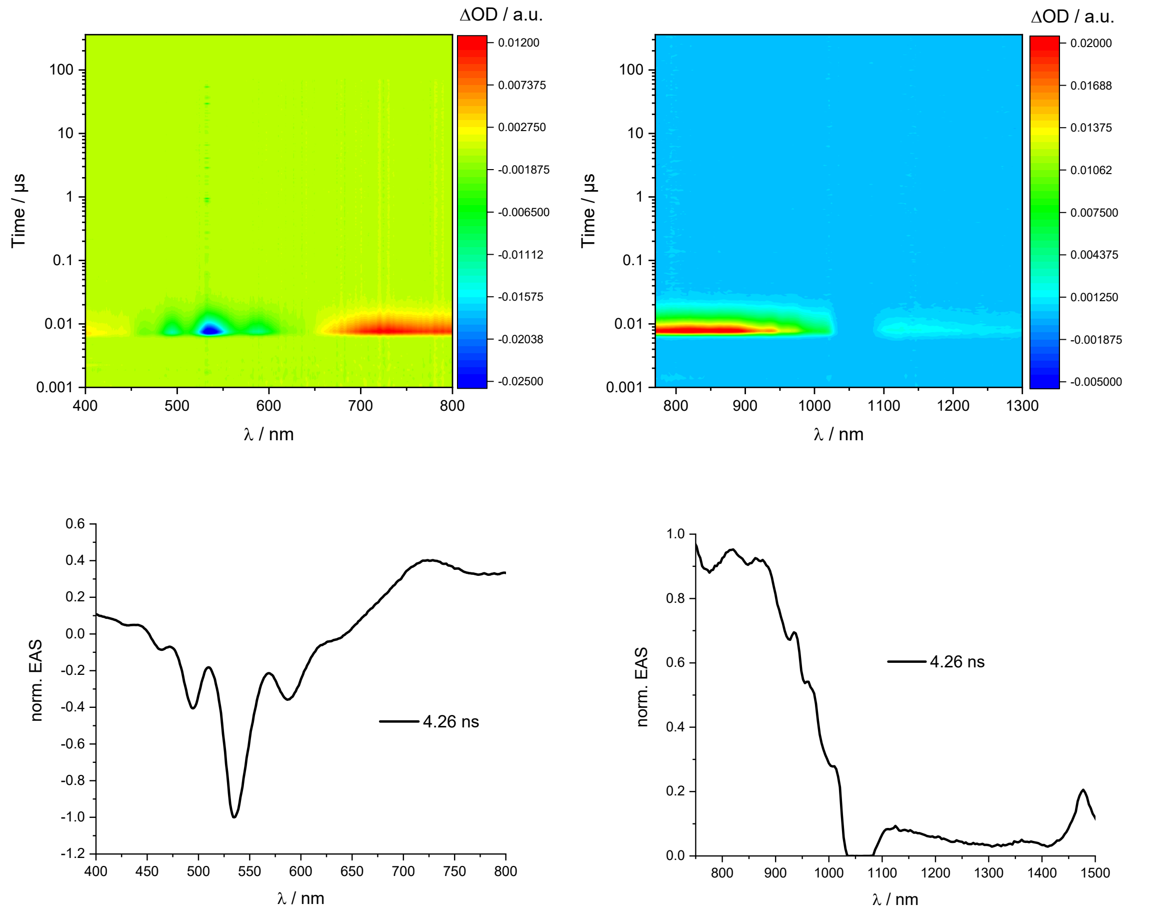

Figure S44. ns-Transient absorption spectroscopy of 1 x 10^{-4} M **7**-QC in THF obtained upon 532 nm photoexcitation with vis detection on the left and NIR detection on the right. Top: 3D plots of the raw data. Bottom: normalized evolution associated spectra obtained by global analysis.

References

(1) Lorenz, P.; Hirsch, A. Photoswitchable Norbornadiene–Quadricyclane Interconversion Mediated by Covalently Linked C60. *Chem. - A Eur. J.* **2020**, *26* (23), 5220–5230. https://doi.org/10.1002/chem.201904679.

(2) Rajasingh, P.; Cohen, R.; Shirman, E.; Shimon, L. J. W.; Rybtchinski, B. Selective Bromination of Perylene Diimides under Mild Conditions. *J. Org. Chem.* **2007**, *72* (16), 5973–5979. https://doi.org/10.1021/jo070367n.

(3) Seger, R.; Mullen, K. M. Glotaran : A Java -Based Graphical User Interface for the R Package TIMP. *J. Stat. Softw.* **2012**, *49* (3), 1–22.

(4) Decke, A. D. Density-Functional Thermochemistry. III. The Role of Exact Exchange. *J. Chem. Phys.* **1993**, *98* (7), 5648–5652.

(5) Yanai, T.; Tew, D. P.; Handy, N. C. A New Hybrid Exchange-Correlation Functional Using the Coulomb-Attenuating Method (CAM-B3LYP). *Chem. Phys. Lett.* **2004**, *393* (1–3), 51–57. https://doi.org/10.1016/j.cplett.2004.06.011.

(6) Boese, A. D.; Martin, J. M. L. Development of Density Functional for Thermochemical Kinetics. *J. Chem. Phys.* **2004**, *121* (8), 3405–3416. https://doi.org/10.1063/1.1774975.

(7) Chai, J. Da; Head-Gordon, M. Long-Range Corrected Hybrid Density Functionals with Damped Atom-Atom Dispersion Corrections. *Phys. Chem. Chem. Phys.* **2008**, *10* (44), 6615–6620. https://doi.org/10.1039/b810189b.

(8) Zhao, Y.; Truhlar, D. G. The M06 Suite of Density Functionals for Main Group Thermochemistry, Thermochemical Kinetics, Noncovalent Interactions, Excited States, and Transition Elements: Two New Functionals and Systematic Testing of Four M06-Class Functionals and 12 Other Function. *Theor. Chem. Acc.* **2008**, *120*, 215–241. https://doi.org/10.1007/s00214-007-0310-x.

(9) An, X.; Xie, Y. Enthalpy of Isomerization of Quadricyclane to Norbornadiene. *Thermochim. Acta* **1993**, *220* (C), 17–25. https://doi.org/10.1016/0040-6031(93)80451-F.

(10) Weigend, F.; Ahlrichs, R. Balanced Basis Sets of Split Valence, Triple Zeta Valence and Quadruple Zeta Valence Quality for H to Rn: Design and Assessment of Accuracy. *Phys. Chem. Chem. Phys.* **2005**, *7* (18), 3297–3305. https://doi.org/10.1039/b508541a.

(11) Weigend, F. Accurate Coulomb-Fitting Basis Sets for H to Rn. *Phys. Chem. Chem. Phys.* **2006**, *8* (9), 1057–1065. https://doi.org/10.1039/b515623h.

(12) Grimme, S.; Ehrlich, S.; Goerigk, L. Effect of the Damping Function in Dispersion Corrected Density Functional Theory. *J. Comput. Chem.* **2011**, *32* (7), 1456–1465. https://doi.org/10.1002/jcc.

Author Contributions

Wiebke Zika

- Shared lead author
- Data acquisition for all spectroscopic experiments
- Formal analysis and investigation of all spectroscopic experiments
- Writing of original draft (spectroscopic sections, Introduction, Conclusion)

Andreas Leng

- Shared lead author
- Synthesis of all mentioned molecules
- Data acquisition for all NMR experiments
- Formal analysis and investigation of all NMR experiments
- Writing of original draft (synthesis and NMR sections)

Simone Pintér

- Supporting author
- Data acquisition of electrochemical, spectroelectrochemical and TCSPC experiments

Christoph M. Schüßlbauer

- Supporting author
- Formal analysis and investigation of electrochemical, spectroelectrochemical and TCSPC data

René Weiß

- Supporting author
- Acquisition and formal analysis of all theoretical calculations

Timothy Clark

- Supporting author
- Supervision of all theoretical calculations

Andreas Hirsch

- Supporting author
- Funding acquisition
- Project administration

Dirk M. Guldi

- Supporting author
- Funding acquisition
- Validation of all spectroscopic and electrochemical interpretations
- Project administration
